# New immune phenotypes for treatment response in high-grade serous ovarian carcinoma patients

**DOI:** 10.3389/fimmu.2024.1394497

**Published:** 2024-06-14

**Authors:** Cecilie Fredvik Torkildsen, Marie Austdal, Anders Hagen Jarmund, Katrin Kleinmanns, Eva Karin Lamark, Elisabeth Berge Nilsen, Ingunn Stefansson, Ragnar Kvie Sande, Ann-Charlotte Iversen, Liv Cecilie Vestrheim Thomsen, Line Bjørge

**Affiliations:** ^1^ Department of Obstetrics and Gynecology, Stavanger University Hospital, Stavanger, Norway; ^2^ Centre for Cancer Biomarkers CCBIO, Department of Clinical Science, University of Bergen, Bergen, Norway; ^3^ Department of Research, Stavanger University Hospital, Stavanger, Norway; ^4^ Department of Clinical and Molecular Medicine, and Centre of Molecular Inflammation Research (CEMIR), Norwegian University of Science and Technology (NTNU), Trondheim, Norway; ^5^ Department of Obstetrics and Gynecology, Haukeland University Hospital, Bergen, Norway; ^6^ Department of Pathology, Haukeland University Hospital, Bergen, Norway; ^7^ Department of Clinical Science, University of Bergen, Bergen, Norway

**Keywords:** ovarian cancer, HGSOC, inflammation, surgery, longitudinal, cytokines, RM-ASCA

## Abstract

Despite advances in surgical and therapeutic approaches, high-grade serous ovarian carcinoma (HGSOC) prognosis remains poor. Surgery is an indispensable component of therapeutic protocols, as removal of all visible tumor lesions (cytoreduction) profoundly improves the overall survival. Enhanced predictive tools for assessing cytoreduction are essential to optimize therapeutic precision. Patients’ immune status broadly reflects the tumor cell biological behavior and the patient responses to disease and treatment. Serum cytokine profiling is a sensitive measure of immune adaption and deviation, yet its integration into treatment paradigms is underexplored. This study is part of the IMPACT trial (NCT03378297) and aimed to characterize immune responses before and during primary treatment for HGSOC to identify biomarkers for treatment selection and prognosis. Longitudinal serum samples from 22 patients were collected from diagnosis until response evaluation. Patients underwent primary cytoreductive surgery or neoadjuvant chemotherapy (NACT) based on laparoscopy scoring. Twenty-seven serum cytokines analyzed by Bio-Plex 200, revealed two immune phenotypes at diagnosis: Immune High with marked higher serum cytokine levels than Immune Low. The immune phenotypes reflected the laparoscopy scoring and allocation to surgical treatment. The five Immune High patients undergoing primary cytoreductive surgery exhibited immune mobilization and extended progression-free survival, compared to the Immune Low patients undergoing the same treatment. Both laparoscopy and cytoreductive surgery induced substantial and transient changes in serum cytokines, with upregulation of the inflammatory cytokine IL-6 and downregulation of the multifunctional cytokines IP-10, Eotaxin, IL-4, and IL-7. Over the study period, cytokine levels uniformly decreased in all patients, leading to the elimination of the initial immune phenotypes regardless of treatment choice. This study reveals distinct pre-treatment immune phenotypes in HGSOC patients that might be informative for treatment stratification and prognosis. This potential novel biomarker holds promise as a foundation for improved assessment of treatment responses in patients with HGSOC. ClinicalTrials.gov Identifier: NCT03378297.

## Introduction

Epithelial ovarian cancer (EOC) is the third most common gynecologic malignant tumor, with high-grade serous ovarian carcinoma (HGSOC) being the most frequent and lethal EOC subtype (1). HGSOC is characterized by a rapid spread of malignant cells throughout the abdominal cavity, and consequently, more than 60% of the patients have advanced disease at diagnosis. More than 80% of patients with advanced disease will experience recurrence and, ultimately, disease-related death (2, 3). When feasible, the preferred primary treatment for advanced HGSOC is surgery followed by chemotherapy (4). The removal of all visible tumor lesions, referred to as complete cytoreductive surgery, is associated with increased overall survival (4). Accordingly, residual tumor after surgery is a negative prognostic factor for survival. Advances in surgical techniques have changed the definition of inoperable tumors. While expansion of the surgical boundaries could be beneficial, it is important to weigh the impact of more advanced surgery on patient morbidity, mortality, and quality of life after treatment. For patients with highly disseminated cancer or high risk for negative outcomes from primary surgery, the implementation of neoadjuvant chemotherapy (NACT) prior to interval surgery has been shown to effectively reduce the tumor burden. As a result, there is a decrease in the required surgical extent, all while maintaining survival rates comparable to those achieved through primary cytoreductive surgery. Moreover, this approach is associated with a lower occurrence of surgical complications.

The precise timing for optimal surgical intervention is under active investigation and inquiry (5–8). An optimal, standardized method for stratifying surgical treatment selection is lacking. A diagnostic laparoscopy-based scoring system, in addition to imaging and clinical examination, is currently considered the most robust option (9). Given that treatment responses and survival differ even in patients with similar stage, grade, and histological features, it becomes evident that the biological behavior of the disease plays a crucial role (10–13).

HGSOC tumors are heterogenous and characterized by somatic mutations of the p53-coding gene *TP53*, frequent dysfunctional homologous recombinant DNA repair mechanisms, and copy number alterations (10, 14–16). The composition of the tumor microenvironment (TME) is increasingly recognized for its significant influence on survival. This effect has been attributed to tumor-infiltrating lymphocytes and regulatory T-cells. Recently, a more comprehensive understanding of the immune TME and the involvement of tumor-induced cytokines have emerged. HGSOC tumors are classified into three immune subtypes: immunologically cold, immune excluded, and inflamed tumors (17). These immune phenotypes share some overlapping characteristics with both the molecular subtypes and the copy number subgroups (10, 14, 16, 18, 19). The complex interplay between cytokines within the TME and the systemic cytokines is both dynamic and intricate. Cytokines have the capability to attract immune cells to the tumor site, either to cause tumor suppression or facilitating tumor growth and invasion (17, 20–23). Systemic cytokines influence and respond to the emergence of cancer-associated symptoms, including fatigue, cachexia, and anemia (24–26). In addition, treatment procedures like surgery can affect cytokine levels and their balance in several ways (27–29). Despite the growing understanding of immune evasion mechanisms, no incorporation of immune response into treatment paradigms has been established.

We have used broad and longitudinal cytokine profiling in serum combined with clinical data to identify the immune adaptations associated with prognosis and treatment selection. This method has successfully been applied in other cancers and in pregnancy to provide detailed information about systemic immune variations, disease-specific responses, and responses to medication (30–37). The existing literature primarily focuses on isolated assessment of individual immune markers at specific time points. Moreover, research has often limited its scope to the examination of specific inflammatory cytokines. Few studies have comprehensively analyzed the effects of EOC and its treatment on cytokine responses (38, 39). This study uses longitudinal serum samples (n = 86) from 22 patients with advanced HGSOC included in the IMPACT trial (NCT03378297). As a result of our exploration of these easily accessible biomarkers, we identified that the patients exhibited two distinct immune phenotypes prior to treatment that appeared to be associated with treatment stratification and prognosis.

## Materials and methods

### Study design

**Figure 1:**
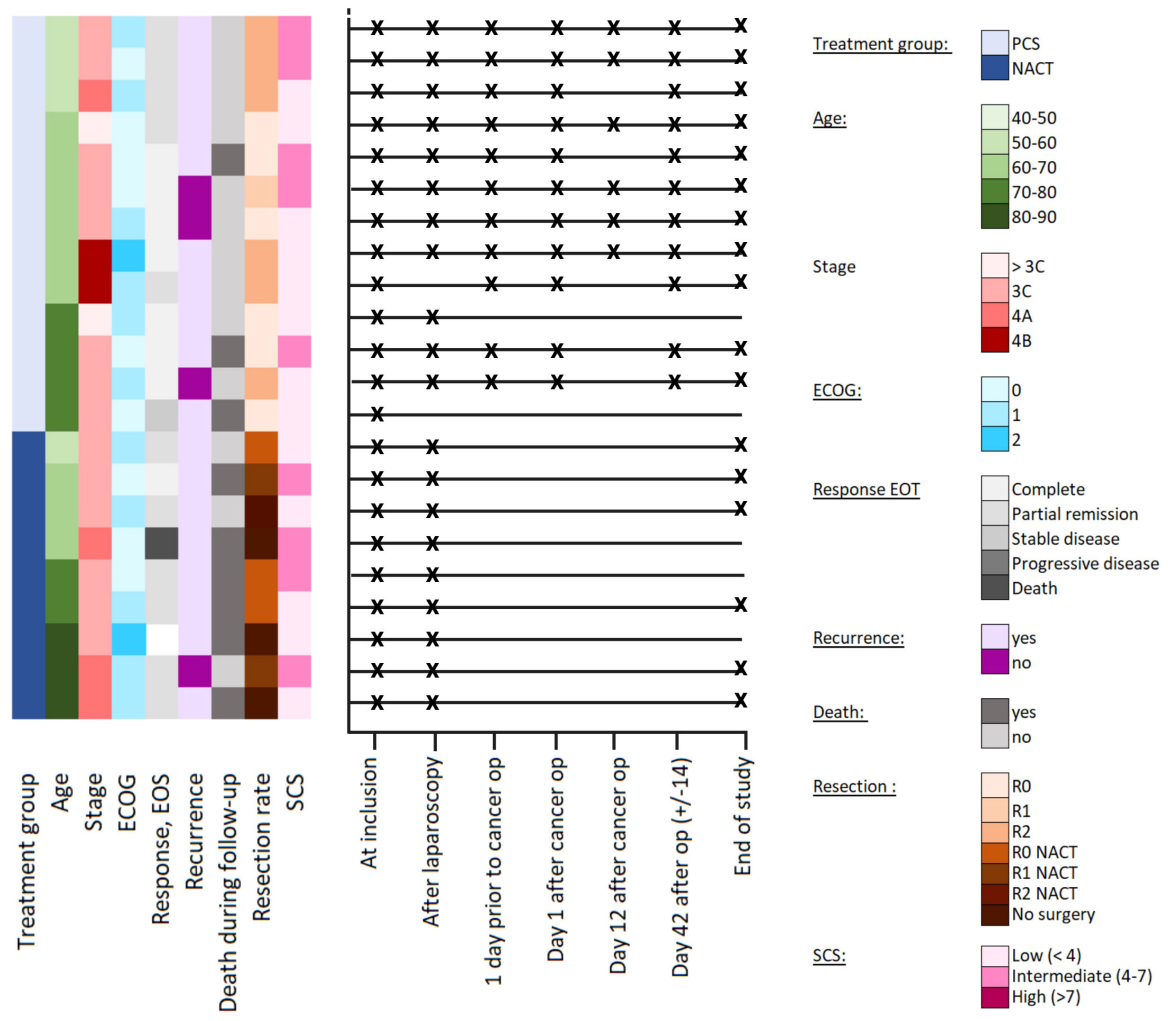
Overview and timeline of the patients. The crosses indicate time points for collecting serum samples; the absence of a cross on the line indicates that serum samples were not obtained at this time point. ECOG, Eastern Cooperative Oncology Group Score; EOS, End of study; NACT, neoadjuvant chemotherapy; PCS, primary cytoreductive surgery; Post-surgery: samples taken 1 day after primary cytoreductive surgery; Pre-chemo: Samples taken day 42 after surgery (+/- 14 days); Pre-surgery: samples taken 1 day prior to primary cytoreductive surgery; R0: Complete cytoreductive surgery; R1: Optimal cytoreductive surgery, residual tumor size ≤1 cm; R2: suboptimal cytoreductive surgery; residual tumor size >1; SCS, surgical complexity score.

The window-of-opportunity IMPACT study (NCT03378297) was a multicenter open-label, single-arm investigation designed to evaluate novel therapeutic strategies between laparoscopy and main cancer surgery (40). Women with advanced (FIGO stage III/IVA) HGSOC were enrolled. All patients underwent a structured diagnostic laparoscopy based on the standardized Predictive Index Value (PIV) upon enrollment in the trial (9, 41). This procedure enabled the allocation of participants into either the primary cytoreductive surgery group (n = 13) or the neoadjuvant chemotherapy group (n = 9). Serum samples were collected at sequential pre-determined time points prior to, during, and after the cancer treatment (**Figure 1**; **Supplementary Figure S1**). Patients allocated to cytoreductive surgery had maximum seven study visits, while those receiving neoadjuvant chemotherapy had three planned study visits.

Progression-free survival (PFS) was calculated as the period between the date of inclusion to the trial and the date of disease progression, either by clinical examination or radiologic imaging. Overall survival was defined as the period between date of inclusion and death of any cause. In addition, the surgical extent was calculated using the standardized surgical complexity score for gynecological malignancies. Complexity scores ≤ 3 are regarded low, 4–7 is intermediate and ≥ 8 is regarded as high surgical complexity (42).

### Ethics and approvals

All subjects provided their written informed consent before inclusion. The clinical trial and the study protocol were approved by the Regional Ethical Committee of Norway (Approval no. 2017/1168) and the Norwegian Medicine Agency (Approval no. 17/10642). The trial was registered at the European Union Drug Regulating Authorities Clinical Trials Database (EudraCT 2017–001689–11) and Clinicaltrials.gov (NCT03378297). The study was conducted in accordance with the Good Clinical Practice guidelines, the Declaration of Helsinki, and local regulations.

### Samples

Peripheral blood was collected in SSTII plus advanced vacutainers, left to clot for 30–90 min at room temperature, centrifuged at 1800 g for 10 min, and the resulting serum aliquots were stored at -80°C until analysis. An overview of the sampling schedule is shown in **Supplementary Figure S1**, and available samples shown in **Figure 1** and **Supplementary Figure S1**. The median number of available samples for cytokine analysis of each patient were n = 6 (range 1–7) in the primary cytoreductive surgery group and n = 3(range 2–3) in the NACT group.

### Cytokine analyses

The serum samples were analyzed for 27 cytokines (with the Bio-Plex Pro Human Cytokine 27-plex Assay) in single replicates using Luminex xMAP Technology on a Bio-Plex 200 System (Bio-Rad Laboratories, CA, USA). The cytokines were classified into four functional groups based on their main function: inflammatory cytokines, anti-inflammatory cytokines, growth- and colony-stimulating factors, and chemokines (**Supplementary Table S1**) (32). The manufacturer’s protocol was followed with the recommended concentration of reagents and serum, but in reduced volumes (1:2) as used in previous studies without compromising the performance of the assay (32–34, 36, 37). The serum samples were randomly distributed across plates using a block design, and each plate contained an interplate serum control and cytokine standards in duplicates. The serum control samples were obtained from two women diagnosed with severe preeclampsia, selected with the expectation of exhibiting positive values across a broad spectrum of cytokines.

### Data preprocessing

Immunoassay is a high-throughput technology in which the results can be influenced by, and need correction for, batch effects to avoid conclusions based on random external events (43). Therefore, the samples were adjusted using fluorescent intensity (FI) values from the serum controls before the concentrations were estimated from standard curves, as previously described (33). The measured values were individually corrected for each cytokine. In brief, the mean log FI value was estimated for the serum control samples on each sample plate and used to adjust the FI value for all the samples on the sample plates. Cytokine standard curves were estimated based on the standard samples from all the sample plates. The nCal package for R was used to calculate concentrations and limit of detection (LOD) values for each cytokine. Non-detectable cytokine values below the lower (L-)LOD were imputed using an expectation-maximization algorithm for the zCompositions package in R (44). Cytokine values above the upper (U-)LOD were replaced by ULOD. Cytokines with values more than 36% above the ULOD or below the LLOD were excluded (IFN-γ, IL-10, RANTES, and VEGF) (**Supplementary Table S1**).

### Statistical analysis

Cytokine concentrations were log-transformed and normalized to ensure concordance of the hazard ratios, and to improve the normality of the residuals before statistical analysis. Visualizations were made with the ggplot2 package in R version 4.0.2 (45, 46).

Differences in clinical variables between patient groups were assessed by *t*-tests for continuous variables and chi-squared tests and ANOVA for categorical variables. Kaplan–Meier curves were used to calculate differences in survival. Changes in serum cytokine levels between visits and between patient groups were modelled with paired *t*-tests and independent samples *t*-tests. Statistical analyses were performed in SPSS v. 29 (SPSS, Chicago, IL, USA) and R. Correction for multiple testing was performed with the Benjamini-Hochberg procedure (47) and adjusted p-values < 0.05 were considered significant.

The changes in serum cytokine levels between the study visits were assessed with linear mixed models (LMMs) using the lme4 package for R (48). Statistical significance was calculated using Satterthwaite’s approximation with the serum levels at the first visit as baseline, unless otherwise stated. Correlations between log-transformed cytokine concentrations were calculated separately, using Spearman’s correlation coefficient (ρ).

Each variable in the cytokine heatmaps was mean centered and variance scaled across samples, so that higher values were indicative of relatively higher concentrations of cytokines among the included samples. Samples were clustered using Euclidian distance and complete linkage, while cytokines were ordered by classification into functional groups based on Stokkeland et al. (32). The heatmaps were constructed using the pheatmap packages in R version 4.0.2 (46).

Repeated-measures ASCA+ (RM-ASCA+) was used to assess longitudinal changes and differences between patient groups (49). RM-ASCA+ is used for analysis of repeated-measures multivariate data that combines traditional univariate statistics of longitudinal data with multivariate dimension reduction techniques. First, a separate LMM was constructed for each cytokine for estimating the effect of time and group and the interaction between time and group for each variable. Second, principal component analysis was performed on the resulting effect matrices, which yielded component scores and loadings for the extraction of patterns across variables. Time, group, time-group interaction, and cohort were included as fixed effects, with the participant as the random intercept. Analyses were performed with the ALASCA package in R using default scaling (sdall) as appropriate (50, 51). Non-parametric bootstrapping was used to construct 95% confidence intervals for the scores and loadings. Bootstrapping was repeated 1,000 times and was performed by resampling until the original sample size was achieved. The 2.5^th^ and 97.5^th^ percentiles of the bootstrapped estimates were used as the lower and upper bounds for the intervals. LMM and RM–ASCA+ analyses were performed in R using the lme4 v1.1–31 (48) and ALASCA v.1.0.0 libraries (51).

## Results

### Clinical and immunological characterization at inclusion

Blood samples from 22 of the 26 patients enrolled in the IMPACT study were assessed (13 treated with primary cytoreductive surgery, and 9 allocated to NACT). Of the original 26 patients, two participants did not meet the inclusion criteria (40). Two patients with autoimmune diseases were excluded from the cytokine analysis because these pre-existing conditions have disease-associated systemic cytokine profiles (52). The descriptive and clinical characteristics of the patients (n = 22) are presented in **Supplementary Table S2**. At the time of data cut-off for the study (March 15^th^, 2023), 19 (86%) patients had experienced relapse and eight (36%) had died. The patient follow-up time varied from 9 to 53 months. The overall survival was significantly higher (p = 0.006) in the primary cytoreductive surgery group than in the NACT group, as previously reported (40). CA125 levels were significantly reduced after tumor-reductive treatment (p < 0.001). Five patients did not complete the treatment (one was excluded due to too low albumin, one due to delayed pathology report, one required an emergency surgery, one died, and one was lost to follow-up) and were not included in the final analysis. This yielded a set of 86 serum samples (22 pre-treatment, 20 after laparoscopy, 11 before primary cytoreductive surgery, 16 between primary cytoreductive surgery and initiation of chemotherapy and 17 end of study samples) (**Figure 1**). These samples were analyzed for cytokines.

**Figure 2:**
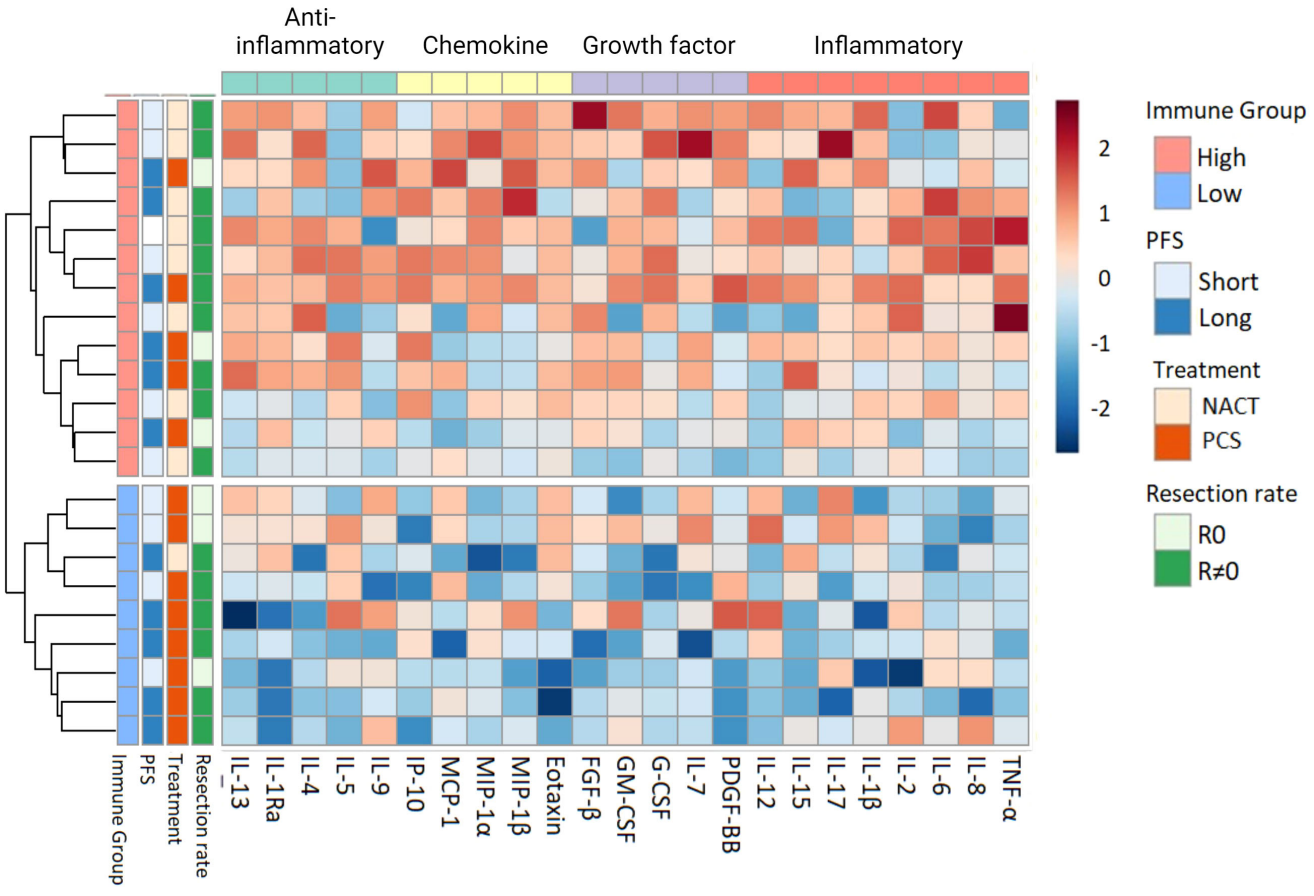
Heatmap with unsupervised hierarchical clustering of serum cytokine levels in all patients (n = 22) at inclusion. Each row represents one patient. The blocks to the left are color coded according to the predefined relevant subgroups (53). Progression-free survival (PFS) was separated into long vs short PFS based on the median value in the dataset (489 days). The color scale represents the serum concentrations of cytokines, with lower concentrations in blue and higher concentrations in red. The color scale is relative and scaled for inter-individual differences between patients for each cytokine. NACT, Neoadjuvant chemotherapy; PCS, primary cytoreductive surgery; PFS, Progression-free survival; R0, Complete cytoreductive surgery; R≠0, Residual tumor after surgery.

Unsupervised hierarchical clustering of the serum cytokine levels at inclusion revealed two distinct immune phenotypes among the patients prior to treatment: The first characterized by markedly higher serum cytokine levels, subsequently referred to as Immune High (n = 13), and the second with lower serum cytokine levels, denoted as Immune Low (n = 9) (**Figure 2**). The serum cytokine levels in Immune High patients indicated substantial immune mobilization, with significantly higher serum levels in approximately half of the examined cytokines (12/23) encompassing all four functional cytokine groups (**Supplementary Table S3**). The descriptive and clinical characteristics for the patient immune groups can be found in **Supplementary Table S2**. Notably, the Immune High patients demonstrated elevated leukocyte and platelet counts compared to the Immune Low patients. (**Supplementary Table S2**). Immune High patients also displayed a trend toward shorter overall survival (p = 0.062), and 62% of them (8/13) were allocated to NACT. Conversely, Immune Low patients were characterized by significantly lower serum cytokine levels before treatment compared to the Immune High group, and all but one patient had been assigned to primary cytoreductive surgery.

### Immune high and immune low patient groups

Following surgical treatment, the Immune High patients displayed a more extensive immune response compared to the Immune Low patients (**Supplementary Table S4**). In a subgroup analysis of patients who underwent primary cytoreductive surgery (Immune High: 5/13, Immune Low: 8/9), it was observed that the Immune High patients more frequently achieved complete cytoreduction compared to the Immune Low group (60% vs 38%, 3/5 vs. 3/8) (**Figure 2**). Regardless of any residual tumor after primary cytoreductive surgery, the Immune High patients exhibited a superior treatment response, of 100% (5/5) long progression-free survival (PFS), while only 50% (4/8) of the Immune Low patients did the same.

### Immune adaptions during primary treatment

**Figure 3:**
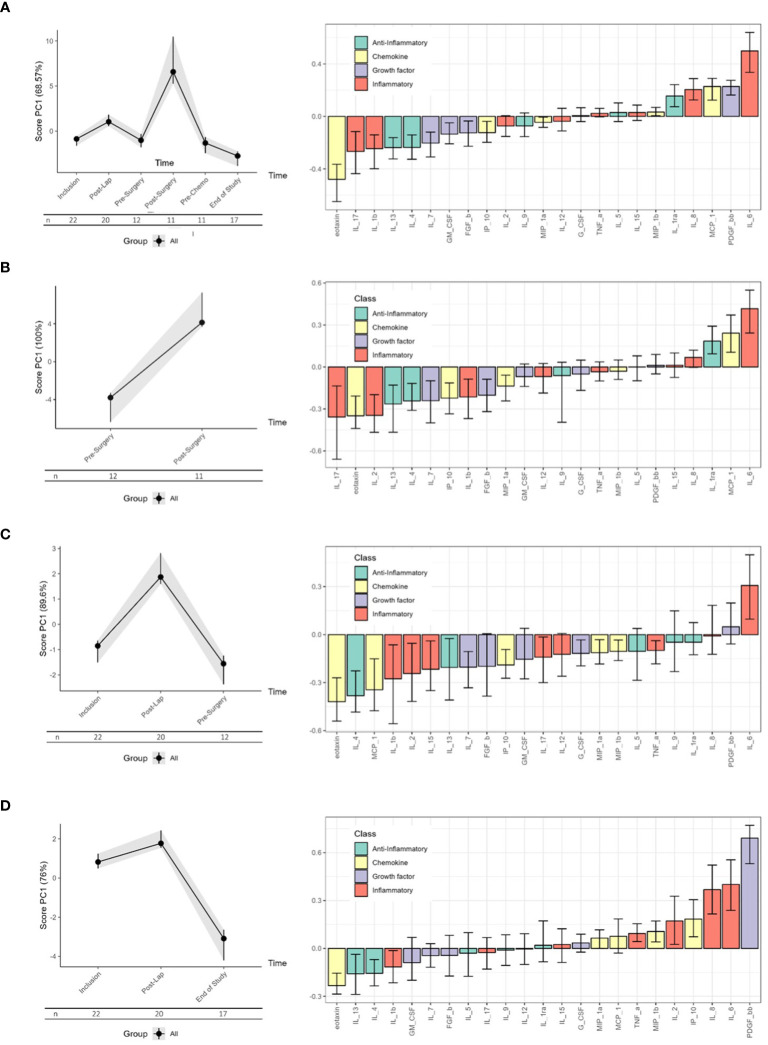
RM-ASCA analyses of longitudinal changes in the serum cytokine levels in all patients (n = 22). The waterfall plots (right panels) illustrate the longitudinal changes in the serum levels of each cytokine according to their contribution to the principal component (PC)1 scores (left panels). Higher scores correspond to decreased levels of cytokines with negative loading and higher levels of cytokines with positive loading. **(A)** All the patients and all the visits are presented (n = 22). **(B)** Changes related to cytoreductive surgery only (n = 11). **(C)** Changes related to laparoscopic surgery only (n = 20). **(D)** Overall treatment-related changes (n = 17). Only visits occurring in both study groups are included. Cytokine functional groups are color-coded as indicated, and black lines within the bars represent the 95% confidence interval.

Longitudinal serum cytokine profiling of all patients throughout the treatment period demonstrated a wide range of immune responses (**Figure 3A**). Based on the results from prior studies on immune responses to surgical interventions, it is evident that several of the observed responses were influenced by the surgical treatment. The most pronounced immunological impact was in response to the primary cytoreductive surgery, as illustrated in **Figure 3A**. Notably, marked changes were identified in 13 of the 23 cytokines across all four functional cytokine groups (**Figure 3B**; **Supplementary Table S5**) immediately after the cytoreductive surgery. This predominantly suppressive cytokine response was transient and had returned to the pre-surgery levels within 14–30 days (**Supplementary Tables S6**, **S7**). The diagnostic laparoscopy also demonstrated a notable and transient influence on the serum cytokine levels (**Figures 3A, C**; **Supplementary Tables S5**, **S8**). The immune adaption to both laparoscopic and cytoreductive surgery showed some overlapping cytokine patterns (**Supplementary Tables S5**, **S9**), characterized by a reduction in the chemokines eotaxin, IP-10 and MCP-1, the anti-inflammatory IL-4, and the growth factor IL-7 (**Figure 3C**; **Supplementary Table S8**). Only the Immune High patients, opposed to the Immune Low patients, exhibited significant alterations in serum cytokine levels following the laparoscopic procedure (**Supplementary Table S3**).

### Overall impact of treatment

**Figure 4:**
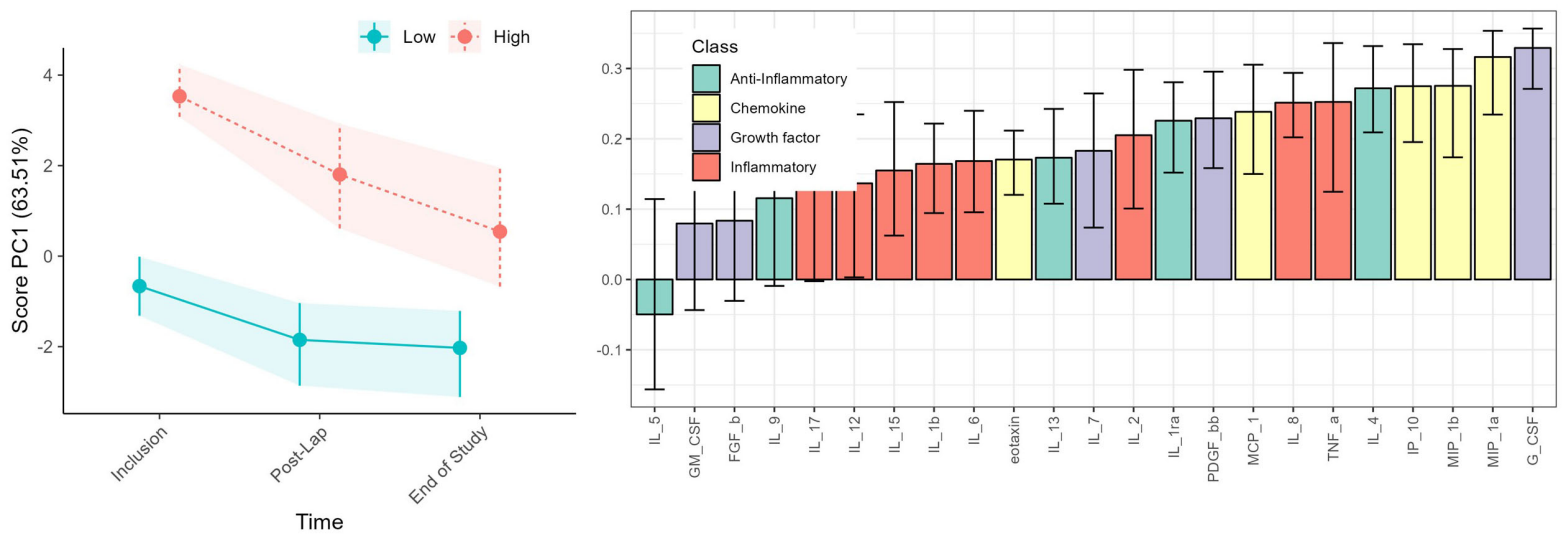
RM-ASCA analysis illustrating cytokine trajectories categorized into Immune High (red) (n = 13) and Immune Low (green) (n = 9) patients. Both time and cytokine level variations are depicted, but the waterfall plots (right) primarily highlight the differences between the patient immune groups as these persists during the study period. Cytokine functional groups are color-coded as indicated, and black lines within the bars represent the 95% confidence interval.

The overall serum cytokine levels declined from inclusion to the end of study (**Figures 3A, D**), together with a concomitant reduction in tumor burden (using the measured fall in the CA125 level as a surrogate marker) (**Supplementary Table S2**). A pronounced and substantial alteration in serum cytokine levels was evident for the Immune High patients, while the overall immune changes in the Immune Low patients was apparent, but markedly lower (**Figure 4**; **Supplementary S10**).

**Figure 5:**
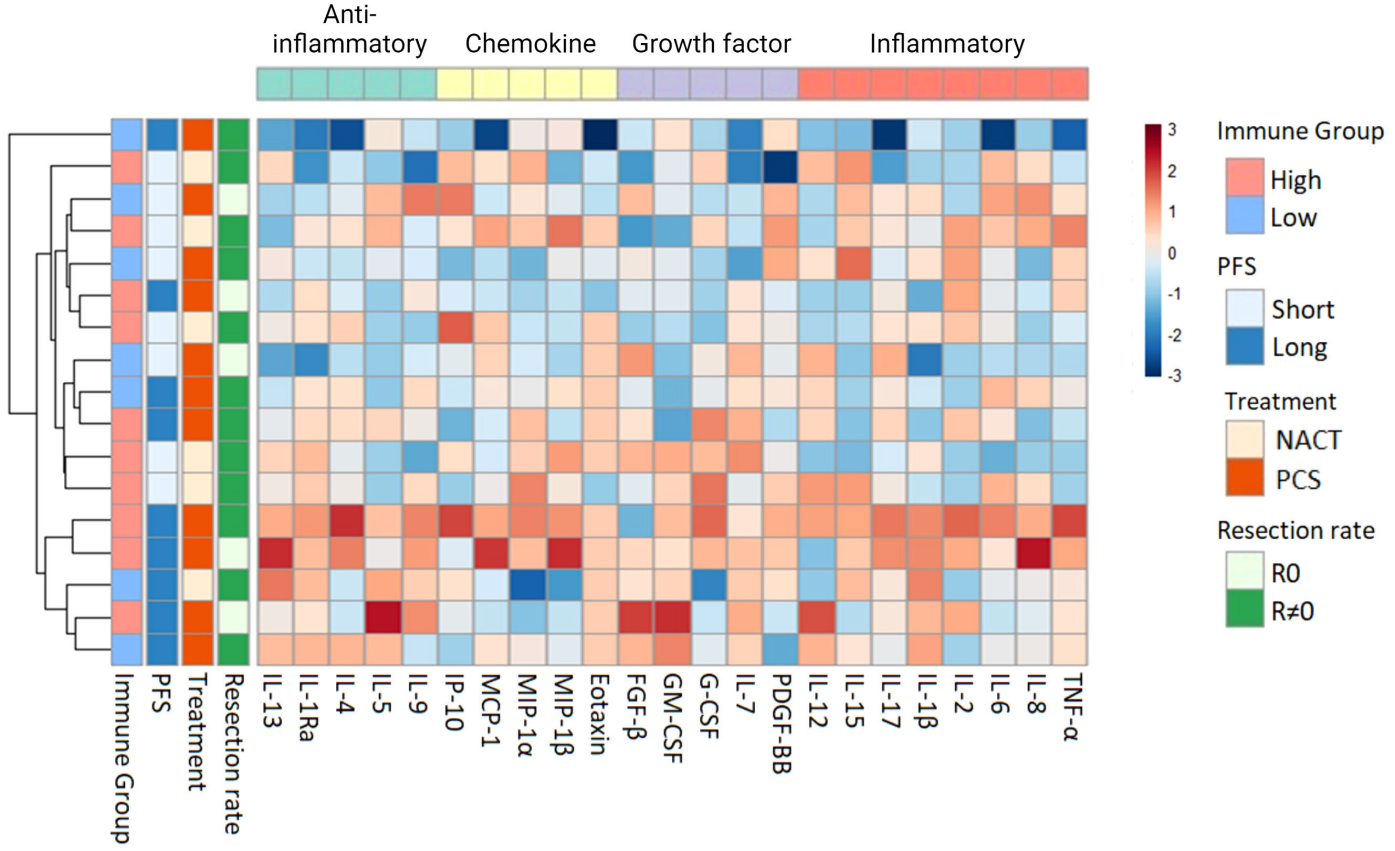
Heatmap with unsupervised hierarchical clustering of all patients (n = 17) at the end of study. Each row represents one patient. The blocks to the left are color coded according to the predefined relevant subgroups (53). The cytokine classes are shown at the top. The color scale represents the serum concentrations of cytokines, with lower concentrations in blue and higher concentrations in red. The color scale is relative and scaled for inter-individual differences between patients for each cytokine. NACT, Neoadjuvant chemotherapy; PCS, Primary cytoreductive surgery; PFS, Progression-free survival; R0, Complete cytoreductive surgery; R≠0, Residual tumor after surgery.

The serum cytokine profile at the end of the study divided the patients into heterogenous groups (**Figure 5**; **Supplementary Table S10**) and the Immune High and Low patient groups apparent at inclusion could no longer be separated (**Figure 2**; **Supplementary Figure S3**). At the end of study visit, the patients with highest serum cytokine levels (lower cluster, n = 5/17) were mainly from the primary cytoreductive surgery group (4/5) and consistently showed an extended time to disease progression. This association persisted regardless of the presence of residual tumor after primary cytoreductive surgery (3/5) or patient immune group prior to treatment (3/5).

## Discussion

This study stans out among its peers for its integration of longitudinal multiplex cytokine profiling alongside clinical attributes of patients diagnosed with HGSOC. Two distinct immune groups were defined prior to treatment initiation: Immune High and Immune Low. Immune High patients had higher serum levels of approximately half the cytokines, including all four functional cytokine groups, than the Immune Low patients. Most Immune High patients received NACT, while almost all patients in the Immune Low group underwent primary cytoreductive surgery. Patients with the Immune High profile prior to treatment demonstrated more advanced tumor dissemination. Further, the findings demonstrated that the surgical procedures induced marked and transient changes in serum cytokine levels. An overall decline in serum cytokine levels was noted at the end of treatment across the entire cohort. Intriguingly, elevated immune activity observed at the end-of-study visit appeared to correlate with prolonged progression-free survival. Nevertheless, this correlation did not attain statistical significance (n = 0.095). The observed sustained alteration in serum cytokine profiles from the time of enrollment over the treatment course and follow-up period resulted in the convergence of the originally defined pre-treatment immune groups and was associated with the concurrent reduction in tumor burden in the entire cohort.

It is difficult to predict the achievable degree of cytoreduction with the currently available preoperative assessment methods. The Fagotti scoring system, which received the approval of participating institutions a year before the IMPACT trial, was implemented as a part of these efforts. Regrettably, only 50% of the patients in the trial achieved complete cytoreduction (40), which was lower than that predicted by the Fagotti scores when the method was introduced and those reported in published studies.

The Immune High and Immune Low profiles represent distinct patterns of immune activation, with the former characterized by a marked and broad immune activated state and the latter, by a more immunologically quiescent pattern. The elevated leukocyte counts in the Immune High patients compared to the Immune Low patients is suggestive of additional activation of the cellular immune system. However, serum cytokine levels are relative values that should be interpreted in conjunction with the clinical outcomes. The reduction in tumor load, illustrated by a significant decline in the tumor marker CA125 (p < 0.001), was observed both after surgery and during chemotherapy. This finding coincided with a decline in cytokine levels towards at the end of treatment. This immune reduction was most evident in the Immune High patients who no longer exhibited a distinct cytokine profile at the end of treatment. Together, these findings suggest that the overall tumor burden and the TME influence the original immune profiles, as described in follicular lymphoma (54).

Another unresolved question that remains to be answered is whether the comparatively elevated cytokine levels observed in the Immune High group are causally linked to symptom development, as suggested by the higher ECOG scores and lower albumin levels in this cohort. Alternatively, they may play a role in disease dissemination, as evidenced by the higher predictive index value (PIV) scores, as previously described in non-small cell lung cancer (55, 56). Cytokine levels could also reflect signals from the TME, inflammation generated by tumor dissemination, or peripheral immune cells. Further investigations are necessary to understand how the identified cytokine signaling profiles correlate with the spatial, temporal, and intra-tumoral heterogeneity of primary tumors and the systemic immune cell composition in peripheral blood in patients with HGSOC.

Surgery elicits an immediate immune response that culminates in postoperative immunosuppression, a phenomenon initiated by surgical intervention, anesthesia, blood transfusion and administration of anti-inflammatory medications (57). Our findings align with this, demonstrating rapid and transient changes in cytokine profiles following both laparoscopy and cytoreductive surgery, with the most pronounced alterations observed after major surgery. The immunological groups exhibited distinct patterns of immune activation after surgical procedures. That is, the Immune Low patients appeared to be immunologically unaffected by diagnostic laparoscopy, whereas the Immune High patients displayed an immune response that was characterized by significant changes in anti-inflammatory and chemokine factors. While the impact of laparoscopic procedures on tumor behavior in both the Immune High and Immune Low patient groups warrants further investigation, it is tempting to speculate that the initiation of a tumor-promoting immunological response, such as the activation seen in the Immune High group, could potentially explain the lower-than-expected prediction rate for successful surgery based on the PIV score in the IMPACT clinical trial. Moreover, we cannot dismiss the possibility that laparoscopy-induced inflammation facilitates further tumor growth and spread. Consequently, we suggest that the time interval between laparoscopy and the primary surgical procedure is reduced to ensure that both procedures are conducted within the same setup for patients with disease dissemination amenable to surgical resection.

Despite cytoreductive surgery’s significant trauma only two cytokine subgroups (that is, anti-inflammatory cytokines and chemokines) were affected in the Immune Low patients while the Immune High patients exhibited a broader response impacting all four functional cytokine groups. Surgery-induced stress not only heightens inflammation but also activates the sympathetic nervous system and potentially further influencing the cytokine response. In patients with EOC, surgical procedures have been shown to elevate the presence of circulating tumor cells, potentially predisposing the patients to distant metastasis either directly or following a period of dormancy (58). Other studies indicate that tumor resection affects the immune control of tumor cells (59, 60). This suggests the potential role of cytokines in the development of metastatic disease, and that this effect is possibly triggered by surgery-related stress.

## Limitations

Limitations of our study are the relatively small cohort size and the lack of validation of the findings in an independent patient cohort. Incorporating a healthy matched control group would have provided valuable context for interpreting immunological effects allowing for a comparative analysis of immunological activation and suppression relative to a baseline immunological status. The IMPACT trial had to be prematurely discontinued due to recruitment challenges arising from the COVID-19 pandemic, leaving relatively small and unevenly distributed patient groups. Sample size calculations for longitudinal studies are not well explored in trials without a fixed exposure, and power calculation was not performed (61). Despite the low number of participants, longitudinal analysis can still yield clinically relevant finding and is more cost-effective than cross-sectional studies, which require larger sample sizes and single timepoint measurements (62). Another limitation pertains to the selection of patients for primary cytoreductive surgery. This discrepancy may be attributed to the learning curves associated with implementing a new stratification tool, although this had been considered when the protocol was designed. The risk of delaying standard-of-care treatment is an inherent limitation to the window-of-opportunity design, but this was minimized by strict inclusion criteria and close monitoring (63).

## Conclusion

Through serum cytokine profiling, we have identified potential new immunophenotypes, delineating two distinct immune profiles characterized by different disease phenotypes and prognoses. The findings indicate a potential advantage of adding broad cytokine profiling to the diagnostic work-up of HGSOC patients for treatment allocation. The longitudinal cytokine patterns demonstrated valuable additional information about the immunological impact of surgery, general cancer treatment, and disease development for these patients. Considering these results and the intricate and dynamic nature of both the immune system and malignancies, this study demonstrates how longitudinal serum analysis offers a highly sensitive tool for exploring disease progression and therapy response. Furthermore, the sequential immune changes in cytokine patters found for the distinct HGSOC patient immune groups should be explored further in larger cohorts. Serum-based analysis can be easily performed without major invasive procedures and serves as a practical source for biomarkers in clinical practice. However, the use of serum cytokine profiling warrants validation before it can be evaluated in prospective trials, and it is also necessary to determine how the cytokine patterns reflects the tumor and the patient´s immune system.

## Data availability statement

Data cannot be made publicly available for ethical reasons. The authors can make the data available upon request with permission of the Norwegian Regional Ethical Committee. Researchers can contact the corresponding author.

## Ethics statement

The studies involving humans were approved by Regional Ethical Committee of Norway (Approval no. 2017/1168) and the Norwegian Medicine Agency (Approval no. 17/10642). The studies were conducted in accordance with the local legislation and institutional requirements. The participants provided their written informed consent to participate in this study.

## Author contributions

CT: Conceptualization, Data curation, Formal analysis, Funding acquisition, Investigation, Methodology, Project administration, Resources, Software, Supervision, Visualization, Writing – original draft, Writing – review & editing. MA: Data curation, Formal analysis, Validation, Visualization, Writing – original draft, Writing – review & editing, Software, Supervision. AJ: Formal analysis, Writing – review & editing. KK: Writing – review & editing. EL: Writing – review & editing, Investigation. EN: Writing – review & editing, Investigation. IS: Investigation, Writing – review & editing. RS: Investigation, Supervision, Writing – review & editing. AI: Data curation, Funding acquisition, Methodology, Writing – original draft, Writing – review & editing. LT: Conceptualization, Data curation, Formal analysis, Funding acquisition, Investigation, Methodology, Project administration, Resources, Supervision, Writing – original draft, Writing – review & editing. LB: Conceptualization, Data curation, Formal analysis, Funding acquisition, Investigation, Methodology, Project administration, Resources, Software, Supervision, Validation, Visualization, Writing – original draft, Writing – review & editing.

## References

[1] Siegel RL Miller KD Fuchs HE Jemal A. Cancer statistics, 2022. CA Cancer J Clin. (2022) 72:7–33. doi: 10.3322/caac.21708 35020204

[2] Borley J Wilhelm-Benartzi C Brown R Ghaem-Maghami S. Does tumour biology determine surgical success in the treatment of epithelial ovarian cancer? A systematic literature review. Brit J Cancer. (2012) 107:1069–74. doi: 10.1038/bjc.2012.376 PMC3461167 22935582

[3] Ushijima K. Treatment for recurrent ovarian cancer-at first relapse. J Oncol. (2010) 2010:497429. doi: 10.1155/2010/497429 20066162 PMC2801501

[4] du Bois A Reuss A Pujade-Lauraine E Harter P Ray-Coquard I Pfisterer J. Role of surgical outcome as prognostic factor in advanced epithelial ovarian cancer: a combined exploratory analysis of 3 prospectively randomized phase 3 multicenter trials: by the Arbeitsgemeinschaft Gynaekologische Onkologie Studiengruppe Ovarialkarzinom (AGO-OVAR) and the Groupe d’Investigateurs Nationaux Pour les Etudes des Cancers de l’Ovaire (GINECO). Cancer. (2009) 115:1234–44. doi: 10.1002/cncr.24149 19189349

[5] Reuss A du Bois A Harter P Fotopoulou C Sehouli J Aletti G. TRUST: Trial of Radical Upfront Surgical Therapy in advanced ovarian cancer (ENGOT ov33/AGO-OVAR OP7). Int J Gynecol Cancer. (2019) 29:1327–31. doi: 10.1136/ijgc-2019-000682 31420412

[6] Fagotti A Ferrandina MG Vizzielli G Pasciuto T Fanfani F Gallotta V. Randomized trial of primary debulking surgery versus neoadjuvant chemotherapy for advanced epithelial ovarian cancer (SCORPION-NCT01461850). Int J Gynecol Cancer. (2020) 30:1657–64. doi: 10.1136/ijgc-2020-001640 33028623

[7] Kehoe S Hook J Nankivell M Jayson GC Kitchener H Lopes T. Primary chemotherapy versus primary surgery for newly diagnosed advanced ovarian cancer (CHORUS): an open-label, randomised, controlled, non-inferiority trial. Lancet. (2015) 386:249–57. doi: 10.1016/S0140-6736(14)62223-6 26002111

[8] Vergote I Coens C Nankivell M Kristensen GB Parmar MKB Ehlen T. Neoadjuvant chemotherapy versus debulking surgery in advanced tubo-ovarian cancers: pooled analysis of individual patient data from the EORTC 55971 and CHORUS trials. Lancet Oncol. (2018) 19:1680–7. doi: 10.1097/01.ogx.0000554460.78765.59 30413383

[9] Fagotti A Ferrandina G Fanfani F Garganese G Vizzielli G Carone V. Prospective validation of a laparoscopic predictive model for optimal cytoreduction in advanced ovarian carcinoma. Am J Obstet Gynecol. (2008) 199:642.e1–6. doi: 10.1016/j.ajog.2008.06.052 18801470

[10] Cancer Genome Atlas Research N. Integrated genomic analyses of ovarian carcinoma. Nature. (2011) 474:609–15. doi: 10.1038/nature10166 PMC3163504 21720365

[11] Geistlinger L Oh S Ramos M Schiffer L LaRue RS Henzler CM. Multiomic analysis of subtype evolution and heterogeneity in high-grade serous ovarian carcinoma. Cancer Res. (2020) 80:4335–45. doi: 10.1158/0008-5472.CAN-20-0521 PMC7572645 32747365

[12] Achimas-Cadariu P Kubelac P Irimie A Berindan-Neagoe I Ruhli F. Evolutionary perspectives, heterogeneity and ovarian cancer: a complicated tale from past to present. J Ovarian Res. (2022) 15:67. doi: 10.1186/s13048-022-01004-1 35659345 PMC9164402

[13] Lheureux S Braunstein M Oza AM. Epithelial ovarian cancer: Evolution of management in the era of precision medicine. CA Cancer J Clin. (2019) 69:280–304. doi: 10.3322/caac.21559 31099893

[14] Verhaak RG Tamayo P Yang JY Hubbard D Zhang H Creighton CJ. Prognostically relevant gene signatures of high-grade serous ovarian carcinoma. J Clin Invest. (2013) 123:517–25. doi: 10.1172/JCI65833 PMC3533304 23257362

[15] Macintyre G Goranova TE De Silva D Ennis D Piskorz AM Eldridge M. Copy number signatures and mutational processes in ovarian carcinoma. Nat Genet. (2018) 50:1262–70. doi: 10.1038/s41588-018-0179-8 PMC6130818 30104763

[16] Tothill RW Tinker AV George J Brown R Fox SB Lade S. Novel molecular subtypes of serous and endometrioid ovarian cancer linked to clinical outcome. Clin Cancer Res. (2008) 14:5198–208. doi: 10.1158/1078-0432.CCR-08-0196 18698038

[17] Kandalaft LE Dangaj Laniti D Coukos G. Immunobiology of high-grade serous ovarian cancer: lessons for clinical translation. Nat Rev Cancer. (2022) 22:640–56. doi: 10.1038/s41568-022-00503-z 36109621

[18] Davoli T Uno H Wooten EC Elledge SJ. Tumor aneuploidy correlates with markers of immune evasion and with reduced response to immunotherapy. Science. (2017) 355(6322):eaaf8399. doi: 10.1126/science.aaf8399 28104840 PMC5592794

[19] Chen DS Mellman I. Elements of cancer immunity and the cancer-immune set point. Nature. (2017) 541:321–30. doi: 10.1038/nature21349 28102259

[20] Hanahan D. Hallmarks of cancer: new dimensions. Cancer Discov. (2022) 12:31–46. doi: 10.1158/2159-8290.CD-21-1059 35022204

[21] Wang X Wang E Kavanagh JJ Freedman RS. Ovarian cancer, the coagulation pathway, and inflammation. J Transl Med. (2005) 3:25. doi: 10.1186/1479-5876-3-25 15969748 PMC1182397

[22] Krockenberger M Dombrowski Y Weidler C Ossadnik M Honig A Hausler S. Macrophage migration inhibitory factor contributes to the immune escape of ovarian cancer by down-regulating NKG2D. J Immunol. (2008) 180:7338–48. doi: 10.4049/jimmunol.180.11.7338 PMC3607742 18490733

[23] Jammal MP Martins-Filho A Silveira TP Murta EF Nomelini RS. Cytokines and prognostic factors in epithelial ovarian cancer. Clin Med Insights Oncol. (2016) 10:71–6. doi: 10.4137/CMO.S38333 PMC4973765 27512342

[24] Bower JE. Cancer-related fatigue: links with inflammation in cancer patients and survivors. Brain Behav Immun. (2007) 21:863–71. doi: 10.1016/j.bbi.2007.03.013 PMC3630796 17543499

[25] Freire PP Fernandez GJ de Moraes D Cury SS Dal Pai-Silva M Dos Reis PP. The expression landscape of cachexia-inducing factors in human cancers. J Cachexia Sarcopenia Muscle. (2020) 11:947–61. doi: 10.1002/jcsm.12565 PMC7432594 32125790

[26] Natalucci V Virgili E Calcagnoli F Valli G Agostini D Zeppa SD. Cancer related anemia: an integrated multitarget approach and lifestyle interventions. Nutrients. (2021) 13(2):482. doi: 10.3390/nu13020482 33535496 PMC7912724

[27] O’Dwyer MJ Owen HC Torrance HD. The perioperative immune response. Curr Opin Crit Care. (2015) 21:336–42. doi: 10.1097/MCC.0000000000000213 26103142

[28] van der Bij GJ Oosterling SJ Beelen RH Meijer S Coffey JC van Egmond M. The perioperative period is an underutilized window of therapeutic opportunity in patients with colorectal cancer. Ann Surg. (2009) 249:727–34. doi: 10.1097/SLA.0b013e3181a3ddbd 19387333

[29] Okholm C Goetze JP Svendsen LB Achiam MP. Inflammatory response in laparoscopic vs. open surgery for gastric cancer. Scand J Gastroenterol. (2014) 49:1027–34. doi: 10.3109/00365521.2014.917698 24852697

[30] Mahboob S Ahn SB Cheruku HR Cantor D Rennel E Fredriksson S. A novel multiplexed immunoassay identifies CEA, IL-8 and prolactin as prospective markers for Dukes’ stages A-D colorectal cancers. Clin Proteomics. (2015) 12:10. doi: 10.1186/s12014-015-9081-x 25987887 PMC4435647

[31] Jabeen S Espinoza JA Torland LA Zucknick M Kumar S Haakensen VD. Noninvasive profiling of serum cytokines in breast cancer patients and clinicopathological characteristics. Oncoimmunology. (2019) 8:e1537691. doi: 10.1080/2162402X.2018.1537691 30713794 PMC6343793

[32] Stokkeland LMT Giskeodegard GF Stridsklev S Ryan L Steinkjer B Tangeras LH. Serum cytokine patterns in first half of pregnancy. Cytokine. (2019) 119:188–96. doi: 10.1016/j.cyto.2019.03.013 30954016

[33] Jarmund AH Giskeodegard GF Ryssdal M Steinkjer B Stokkeland LMT Madssen TS. Cytokine patterns in maternal serum from first trimester to term and beyond. Front Immunol. (2021) 12:752660. doi: 10.3389/fimmu.2021.752660 34721426 PMC8552528

[34] Tangeras LH Austdal M Skrastad RB Salvesen KA Austgulen R Bathen TF. Distinct first trimester cytokine profiles for gestational hypertension and preeclampsia. Arterioscler Thromb Vasc Biol. (2015) 35:2478–85. doi: 10.1161/ATVBAHA.115.305817 26404486

[35] Tangeras LH Stodle GS Olsen GD Leknes AH Gundersen AS Skei B. Functional Toll-like receptors in primary first-trimester trophoblasts. J Reprod Immunol. (2014) 106:89–99. doi: 10.1016/j.jri.2014.04.004 24933117

[36] Stokkeland LMT Giskeodegard GF Ryssdal M Jarmund AH Steinkjer B Madssen TS. Changes in serum cytokines throughout pregnancy in women with polycystic ovary syndrome. J Clin Endocrinol Metab. (2022) 107:39–52. doi: 10.1210/clinem/dgab684 34529073 PMC8684459

[37] Ryssdal M Vanky E Stokkeland LMT Jarmund AH Steinkjer B Lovvik TS. Immunomodulatory effects of metformin treatment in pregnant women with PCOS. J Clin Endocrinol Metab. (2023) 108(9):e743–53. doi: 10.1210/clinem/dgad145 PMC10438881 36916886

[38] Lippitz BE. Cytokine patterns in patients with cancer: a systematic review. Lancet Oncol. (2013) 14:e218–28. doi: 10.1016/S1470-2045(12)70582-X 23639322

[39] Wieder-Huszla S Chudecka-Glaz A Gutowska I Karakiewicz B Jurczak A. Effect of the treatment stage on the serum levels of selected cytokines and antioxidant enzymes in patients with tumors of the reproductive organs. Eur Rev Med Pharmacol Sci. (2023) 27:3117–33. doi: 10.26355/eurrev_202304_31946z 37070915

[40] Torkildsen CF Austdal M Iversen AC Bathen TF Giskeodegard GF Nilsen EB. Primary treatment effects for high-grade serous ovarian carcinoma evaluated by changes in serum metabolites and lipoproteins. Metabolites. (2023) 13(3):417. doi: 10.3390/metabo13030417 36984856 PMC10053757

[41] Fagotti A Ferrandina G Fanfani F Ercoli A Lorusso D Rossi M. A laparoscopy-based score to predict surgical outcome in patients with advanced ovarian carcinoma: a pilot study. Ann Surg Oncol. (2006) 13:1156–61. doi: 10.1245/ASO.2006.08.021 16791447

[42] Aletti GD Podratz KC Moriarty JP Cliby WA Long KH. Aggressive and complex surgery for advanced ovarian cancer: an economic analysis. Gynecol Oncol. (2009) 112:16–21. doi: 10.1016/j.ygyno.2008.10.008 19027146

[43] Leek JT Scharpf RB Bravo HC Simcha D Langmead B Johnson WE. Tackling the widespread and critical impact of batch effects in high-throughput data. Nat Rev Genet. (2010) 11:733–9. doi: 10.1038/nrg2825 PMC3880143 20838408

[44] Palarea-Albaladejo J Martin-Fernandez JA. zCompositions - R Package for multivariate imputation of left-censored data under a compositional approach. Chemometr Intell Lab. (2015) 143:85–96. doi: 10.1016/j.chemolab.2015.02.019

[45] R Core Team. R: A language and environment for statistical computing Vol. 2020. Vienna, Australia: R Foundation for Statistical Computing (2020).

[46] Allen M Poggiali D Whitaker K Marshall T van Langen J Kievit R. Raincloud plots: a multi-platform tool for robust data visualization [version 2; peer review: 2 approved]. Wellcome Open Res. (2021) 4. doi: 10.12688/wellcomeopenres PMC6480976 31069261

[47] Benjamini Y Hochberg Y. Controlling the false discovery rate - a practical and powerful approach to multiple testing. J R Stat Soc B. (1995) 57:289–300. doi: 10.1111/j.2517-6161.1995.tb02031.x

[48] Bates D Machler M Bolker BM Walker SC. Fitting linear mixed-effects models using lme4. J Stat Software. (2015) 67:1–48. doi: 10.18637/jss.v067.i01

[49] Madssen TS Giskeodegard GF Smilde AK Westerhuis JA. Repeated measures ASCA+ for analysis of longitudinal intervention studies with multivariate outcome data. PloS Comput Biol. (2021) 17:e1009585. doi: 10.1371/journal.pcbi.1009585 34752455 PMC8604364

[50] Timmerman ME Hoefsloot HC Smilde AK Ceulemans E. Scaling in ANOVA-simultaneous component analysis. Metabolomics. (2015) 11:1265–76. doi: 10.1007/s11306-015-0785-8 PMC4559107 26366136

[51] Jarmund AH Madssen TS Giskeodegard GF. ALASCA: An R package for longitudinal and cross-sectional analysis of multivariate data by ASCA-based methods. Front Mol Biosci. (2022) 9:962431. doi: 10.3389/fmolb.2022.962431 36387276 PMC9645785

[52] Moudgil KD Choubey D. Cytokines in autoimmunity: role in induction, regulation, and treatment. J Interferon Cytokine Res. (2011) 31:695–703. doi: 10.1089/jir.2011.0065 21942420 PMC3189547

[53] Guida F Dioun S Fagotti A Melamed A Grossi A Scambia G. Role of tertiary cytoreductive surgery in recurrent epithelial ovarian cancer: Systematic review and meta-analysis. Gynecol Oncol. (2022) 166:181–7. doi: 10.1016/j.ygyno.2022.04.005 35550711

[54] Mozas P Rivas-Delgado A Rivero A Dlouhy I Nadeu F Balague O. High serum levels of IL-2R, IL-6, and TNF-alpha are associated with higher tumor burden and poorer outcome of follicular lymphoma patients in the rituximab era. Leuk Res. (2020) 94:106371. doi: 10.1016/j.leukres.2020.106371 32473488

[55] Wang XS Shi Q Williams LA Mao L Cleeland CS Komaki RR. Inflammatory cytokines are associated with the development of symptom burden in patients with NSCLC undergoing concurrent chemoradiation therapy. Brain Behav Immun. (2010) 24:968–74. doi: 10.1016/j.bbi.2010.03.009 PMC2897921 20353817

[56] Kartikasari AER Huertas CS Mitchell A Plebanski M. Tumor-induced inflammatory cytokines and the emerging diagnostic devices for cancer detection and prognosis. Front Oncol. (2021) 11:692142. doi: 10.3389/fonc.2021.692142 34307156 PMC8294036

[57] Tang F Tie Y Tu C Wei X. Surgical trauma-induced immunosuppression in cancer: Recent advances and the potential therapies. Clin Transl Med. (2020) 10:199–223. doi: 10.1002/ctm2.24 32508035 PMC7240866

[58] Kim M Suh DH Choi JY Bu J Kang YT Kim K. Post-debulking circulating tumor cell as a poor prognostic marker in advanced stage ovarian cancer: A prospective observational study. Med (Baltimore). (2019) 98:e15354. doi: 10.1097/MD.0000000000015354 PMC6531062 31096435

[59] Hiam-Galvez KJ Allen BM Spitzer MH. Systemic immunity in cancer. Nat Rev Cancer. (2021) 21:345–59. doi: 10.1038/s41568-021-00347-z PMC8034277 33837297

[60] Horowitz M Neeman E Sharon E Ben-Eliyahu S. Exploiting the critical perioperative period to improve long-term cancer outcomes. Nat Rev Clin Oncol. (2015) 12:213–26. doi: 10.1038/nrclinonc.2014.224 PMC5497123 25601442

[61] Basagana X Xiaomei L Spiegelman D. Power and sample size calculations for longitudinal studies estimating a main effect of a time-varying exposure. Stat Methods Med Res. (2011) 20:471–87. doi: 10.1177/0962280210371563 PMC3777279 20547587

[62] Caruana EJ Roman M Hernandez-Sanchez J Solli P. Longitudinal studies. J Thorac Dis. (2015) 7:E537–40. doi: 10.3978/j.issn.2072-1439.2015.10.63 PMC4669300 26716051

[63] Ursprung S Mossop H Gallagher FA Sala E Skells R Sipple JAN. The WIRE study a phase II, multi-arm, multi-centre, non-randomised window-of-opportunity clinical trial platform using a Bayesian adaptive design for proof-of-mechanism of novel treatment strategies in operable renal cell cancer - a study protocol. BMC Cancer. (2021) 21:1238. doi: 10.1186/s12885-021-08965-4 34794412 PMC8600815

