# Supplementary material for: New immune phenotypes for treatment response in high-grade serous ovarian carcinoma patients
Source: Front Immunol. 2024 Jun 14;15:1394497. doi: 10.3389/fimmu.2024.1394497 (PMC11211251; doi:10.3389/fimmu.2024.1394497)
Supplement: Supplementary file 1 [file DataSheet_1.docx]

Supplementary Material

New immune phenotypes for treatment response in high-grade serous ovarian carcinoma patients

Cecilie Fredvik Torkildsen, Marie Austdal, Anders Hagen Jarmund, Katrin Kleinmanns, Eva Karin Lamark, Elisabeth Berge Nilsen, Ingunn Stefansson, Ragnar Kvie Sande, Ann-Charlotte Iversen, Liv Cecilie Vestrheim Thomsen, Line Bjørge

*** Correspondence:** Cecilie Fredvik Torkildsen: cecilie.torkildsen@uib.no

**Supplementary Table S1:** Overview of the cytokines measured in the 27-plex assay and their main functional group.

| Inflammatory cytokines | Anti-inflammatory cytokines | Growth factors | Chemokines |
| --- | --- | --- | --- |
| IL-1β | IL-1Ra | FGF-β (FGF2) | Eotaxin (CCL11) |
| IL-2 | IL-4 | G-CSF | IP-10 (CXCL10) |
| IL-6 | IL-5 | GM-CSF | MCP-1 (CCL2) |
| IL-8 (CXCL8) | IL-9 | IL-7 | MIP-1α (CCL3) |
| IL-12 | IL-13 | PDGF-BB | MIP-1β (CCL4) |
| IL-15 |  |  |  |
| IL-17 |  |  |  |
| TNF-α |  |  |  |
| *IFN-γ* | *IL-10 (C SIF)* | *VEGF-A* | *RANTES (CCL5)* |

Cytokines with more than 36 % of the measured values above (RANTES only) or below the limit of detection are shown in italics. Alternative cytokine names are enclosed in round brackets.

| **Supplementary Table S2:** Clinical characteristics of the patients with HGSOC at inclusion (visit 1) (n = 22). Patient cohort: All patients underwent a structured diagnostic laparoscopy, and the standardized Predictive Index Value (PIV) scoring (9, 41) upon enrollment in the trial. This procedure enabled the allocation of participants into either the primary cytoreductive surgery group (PIV < 8, n = 13) or the neoadjuvant chemotherapy group (PIV ≥ 8) | **All patients**  **(n = 22)** | **Primary cytoreductive surgery**  **(n = 13)** | **Neoadjuvant chemotherapy**  **(n = 9)** | **p-value**  **(Surgery vs NACT)** | **Immune High**  **(n = 13)** | **Immune Low**  **(n = 9)** | **p-value**  **(High vs Low)** |
| --- | --- | --- | --- | --- | --- | --- | --- |
| Age at diagnosis (years) | 68 (54–85) | 65 (54–78) | 73 (56–85) | 0.061 | 71 (56–85) | 65 (54–81) | 0.156 |
| Stage (FIGO 2014)  Stage 2  Stage 3  Stage 4 | 1  15  6 | 1  9  3 | 0  6  3 | 0.318 | 0  10  3 | 1  5  3 | 0.747 |
| gBRCA mut (% tested) | 0 (95%) | 0 (100%) | 0 (89%) |  | 0 | 0 |  |
| sBRCA mut (% tested) | 0 (50%) | 0 (62%) | 0 (33%) |  | 0 | 0 |  |
| NACT | 9 | 0 | 9 |  | 8 | 1 | *0.031* |
| Primary cytoreductive surgery  R0  R1  R2 | 13/22 | 6  1  6 | NA  NA  NA |  | 5/13  3  0  2 | 8/9  3  1  4 |  |
| BMI (m^2^/kg) | 24 (18–32) | 25 (21–32) | 23 (18–27) | 0.307 | 23 (18–30) | 26 (21–32) | 0.121 |
| CA125 (kU/L) before treatment | 890 (34–2332) | 922 (34–2332) | 805 (138–1372) | 0.776 | 741 (138–1653) | 1066 (34-2332) | 0.287 |
| CA125 (kU/L) end of study (n = 17) | 39 (5–293) | 21 (5–84) | 72 (19–293) | 0.146 | 44 (5–193) | 32 (8–84) | 0.689 |
| Comorbidities (n)  None  Cardiovascular  Other | 7  5  10 | 6  2  5 | 1  3  5 | 0.165 | 4  4  5 | 3  1  5 | 1.000 |
| Cholesterol-reducing drug (%)  Yes  No | 5  17 | 3  10 | 2  7 | 0.684 | 4  9 | 1  8 | 0.360 |
| Albumin concentration at inclusion (g/L) | 38.0 (24.4–47.0) | 39.3 | 36.2 | 0.313 | 36.6 (24.4–46.0) | 40.1 (25.4–47.0) | 0.298 |
| Hemoglobin concentration at inclusion (g/dL) | 13.0 (10.5–14.5) | 13.6 | 12.2 | 0.006 | 12.5 (10.5–14.3) | 13.7 (12.5–14.5) | 0.022 |
| Platelet count at inclusion (10^9^/L) | 390 (183–728) | 340 | 461 | 0.080 | 448 (198–728) | 306 (183–483) | *0.036* |
| Leucocyte count at inclusion (10^9^/L) | 7.20 (3.8–12.5) | 6.74 | 7.86 | 0.230 | 7.9 (4.9–12.5) | 6.2 (3.8–8.4) | 0.051 |
| PIV score | 5 (0–12) | 3 (0-8) | 9 (6-12) | *<0.001* | 7 (2–12) | 3 (0–10) | *0.041* |
| Surgical complexity score | 3.1 (0–7) | 4.2 (2-7) | 1.4 (0-3) | *<0.001* | 2.5 (0–6) | 3.9 (2–7) | 0.090 |
| ECOG (median) at inclusion | 1 (0–2) | 0 (0-1) | 1 (0-2) | *0.011* | 1 (0–2) | 0 (0–1) | 0.071 |
| Evaluation^¤^ (end of study)  Complete response  Partial response  Stable disease  N/A  Death | 8  11  1  1  1 | 7  5  1  0  0 | 1  6  0  1  1 | 0.088 | 5  6  0  1  1 | 3  5  1  0  0 | 0.896 |
| PFS (n reached)  no. of months (95% CI) | 19  20.5 (15–26) | 10  24.2 (17–31) | 9  14.6 (8–21) | *0.012* | 20.3 (13–28) | 20.1 (14–26) | 0.922 |
| OS (n reached)  No. of months (95% CI) | 8  36.5 (30–43) | 2  44 (39–49) | 6  24 (15–34) | *0.009* | 31.5 (23–40) | 41.8 (36–48) | 0.062 |

Data are shown as the number of patients in the respective groups or as mean (min–max).

FIGO 2014: The International Federation of Gynecology and Obstetrics staging consensus from 2014, gBRCA mut: genetic BRCA 1/2 mutation, sBRCA mut: somatic BRCA 1/2 mutation, R0: complete cytoreductive surgery (no residual tumor tissue after surgery), R1: optimal cytoreductive surgery (residual tumor ≤ 1 cm), R2: suboptimal cytoreductive surgery (residual tumor > 2 cm), BMI: body mass index ECOG: Eastern Cooperative Oncology Group performance status, PIV score: Predictive index value, PFS: progression-free survival, OS: overall survival, NA: not applicable, NACT: neoadjuvant chemotherapy. P-values ≤0.05 is considered significant. Significant values are indicated in italic font.

^¤^RECIST criteria supplemented by CA125 response and progression criteria developed by the Gynecologic Cancer InterGroup. CA125 progression has been integrated with objective criteria into a composite definition of progression that is often used in the frontline setting.

**Supplementary** **Table S3:** Overview of significant changes in serum cytokine concentrations between visits and between the Immune High patients (A) and the Immune Low patients (B)

| **Cytokine group** | **Cytokine** | **At inclusion (visit 1)**  **(n = 22)** | **After laparoscopy (visit 2) (n = 20)** | | | | **At the end of study (visit 7) (n = 17)** | | | |
| --- | --- | --- | --- | --- | --- | --- | --- | --- | --- | --- |
|  |  | *A vs B^a^* | *A vs B^a^* | Compared to inclusion (visit 1) | | | *A vs B^a^* | Compared to inclusion (visit 1) | | |
|  |  |  |  | *Total cohort* | *Changes in A* | *Changes in B* |  | *Total cohort* | *Changes in A* | *Changes in B* |
| Anti-inflammatory cytokines | IL1Ra | 0.029 | 0.055* | ns | ns | ns | ns | Ns | ns | ns |
|  | IL-4 | 0.005 | 0.048 | >0.001 | 0.014 | 0.145* | ns | Ns | ns | ns |
|  | IL-5 | ns | ns | ns | ns | Ns | ns | Ns | ns | ns |
|  | IL-9 | ns | ns | ns | ns | Ns | ns | Ns | ns | ns |
|  | IL-13 | 0.036 | 0.068* | ns | ns | Ns | ns | Ns | ns | ns |
| Chemokines | IP-10 | 0.005 | 0.064* | 0.001 | 0.013 | 0.145* | ns | 0.003 | 0.006 | ns |
|  | MIP-1α | 0.018 | 0.083* | ns | ns | Ns | ns | 0.058* | ns | ns |
|  | MCP-1 | ns | ns | 0.049 | 0.145* | Ns | ns | 0.020 | ns | ns |
|  | MIP-1β | 0.025 | 0.090* | ns | ns | Ns | ns | 0.012 | 0.013 | ns |
|  | Eotaxin | 0.075* | ns | <0.001 | 0.01 | 0.071* | ns | Ns | ns | ns |
| Growth factors | FGF- β | 0.025 | ns | Ns | ns | Ns | ns | Ns | ns | ns |
|  | GM-CSF | ns | ns | ns | ns | Ns | ns | Ns | ns | ns |
|  | PDGF-BB | ns | 0.099* | Ns | ns | Ns | ns | <0.001 | <0.001 | 0.062* |
|  | G-CSF | 0.005 | ns | ns | ns | Ns | 0.267* | Ns | Ns | ns |
|  | IL-7 | ns | ns | 0.008 | 0.114* | 0.145* | ns | Ns | Ns | ns |
| Inflammatory cytokines | IL-12 | ns | 0.056* | ns | ns | Ns | ns | Ns | Ns | ns |
|  | IL-17 | ns | ns | ns | ns | Ns | ns | Ns | Ns | ns |
|  | IL-15 | 0.024 | ns | 0.124* | ns | Ns | ns | Ns | 0.034 | ns |
|  | IL-2 | ns | ns | ns | ns | Ns | 0.267* | 0.011 | Ns | 0.187* |
|  | TNF-α | 0.027 | 0.048 | ns | ns | Ns | ns | 0.009 | 0.022 | ns |
|  | IL-1β | 0.026 | ns | 0.064* | 0.085* | Ns | ns | Ns | Ns | ns |
|  | IL-6 | 0.023 | ns | 0.010* | Ns | Ns | Ns | 0.006 | 0.013 | Ns |
|  | IL8 | 0.066* | 0.064* | Ns | Ns | Ns | Ns | 0.002 | 0.020 | 0.243* |

Log-transformed values were used to determine the significance of differences. P-values with 95 % confidence intervals in parenthesis are shown, and were calculated by student’s *t*-test, paired where appropriate, and subjected to multiple testing correction.

ns: not significant. For all significant changes in cytokines, the values for the Immune High group (A) were higher than those for the Immune Low group (B). Cytokines without significantly different values have been removed from the table (IL-17, G-CSF, IL-5, IL-9, and FGF-β). *Changes were nominally significant (before correction for multiple testing). P-values ≤0.05 is considered significant. Significant values are indicated in italic font.

**Supplementary Table S4:** Changes in cytokine levels between visit3 and visit 4 (before and after cytoreductive surgery) in the Immune High subgroup (n = 5) and the Immune Low subgroup (n = 6)

| Group | Cytokine | Immune High subgroup (n=5) | | | | Immune Low subgroup (n=6) | | | |
| --- | --- | --- | --- | --- | --- | --- | --- | --- | --- |
|  |  | p-value (unadjusted) | p-value (adj. BH) | Mean change | SD | p-value (unadjusted) | p-value (adj. BH) | Mean change | SD |
| Inflammatory cytokines | IL-1β | 0.008 | *0.027* | -1.03 | 0.47 | 0.159 | 0.281 | -0.31 | 0.47 |
|  | IL-2 | 0.031 | 0.066 | -1.28 | 0.88 | 0.006 | *0.037* | -1.21 | 0.66 |
|  | IL-6 | 0.002 | *0.019* | 2.68 | 0.88 | 0.000 | *0.009* | 3.70 | 1.08 |
|  | IL8 | 0.199 | 0.229 | 0.24 | 0.35 | 0.337 | 0.554 | 0.29 | 0.67 |
|  | IL-12 | 0.075 | 0.124 | -1.56 | 1.46 | 0.761 | 0.921 | 0.19 | 1.45 |
|  | IL-15 | 0.778 | 0.778 | -0.22 | 1.66 | 0.489 | 0.741 | 0.56 | 1.83 |
|  | IL-17 | 0.099 | 0.135 | -1.23 | 1.28 | 0.054 | 0.124 | -0.58 | 0.57 |
|  | TNF-α | 0.106 | 0.135 | -0.29 | 0.31 | 0.695 | 0.888 | 0.05 | 0.27 |
| Anti-inflammatory cytokines | IL1Ra | 0.034 | 0.066 | 0.98 | 0.69 | 0.024 | 0.080 | 2.32 | 1.78 |
|  | IL-4 | 0.007 | *0.027* | -0.90 | 0.40 | 0.070 | 0.146 | -0.33 | 0.35 |
|  | IL-5 | 0.460 | 0.504 | -0.35 | 0.95 | 0.896 | 0.935 | -0.06 | 1.11 |
|  | IL-9 | 0.100 | 0.135 | -0.55 | 0.58 | 0.807 | 0.928 | 0.07 | 0.70 |
|  | IL-13 | 0.001 | *0.016* | -0.98 | 0.27 | 0.033 | 0.091 | -0.62 | 0.52 |
| Growth factors | FGF-β | 0.012 | *0.027* | -0.41 | 0.21 | 0.036 | 0.091 | -0.46 | 0.40 |
|  | G-CSF | 0.011 | *0.027* | -0.55 | 0.28 | 0.515 | 0.741 | 0.18 | 0.64 |
|  | GM-CSF | 0.074 | 0.124 | -0.75 | 0.70 | 0.935 | 0.935 | -0.02 | 0.50 |
|  | IL-7 | 0.004 | *0.025* | -0.91 | 0.35 | 0.013 | 0.062 | -0.45 | 0.29 |
|  | PDGF-BB | 0.732 | 0.765 | -0.04 | 0.21 | 0.573 | 0.776 | 0.11 | 0.46 |
| Chemokines | Eotaxin | 0.011 | *0.027* | -0.82 | 0.41 | 0.004 | *0.030* | -0.61 | 0.30 |
|  | IP-10 | 0.000 | *0.002* | -1.02 | 0.15 | 0.002 | *0.024* | -0.65 | 0.27 |
|  | MCP-1 | 0.083 | 0.127 | 0.44 | 0.43 | 0.017 | 0.067 | 0.70 | 0.49 |
|  | MIP-1α | 0.009 | *0.027* | -0.70 | 0.33 | 0.078 | 0.149 | -0.34 | 0.37 |
|  | MIP-1β | 0.114 | 0.138 | -0.17 | 0.19 | 0.873 | 0.935 | 0.01 | 0.20 |

Paired *t*-test for differences between the pre-surgery visit and the post-surgery visit (1 ± 1 days after surgery). Adj. BH = P-value adjusted with the Benjamini-Hochberg method. P-values ≤0.05 is considered significant. Significant values are indicated in italic font.

**Supplementary Table S5:** Changes in cytokine levels between visit 3 and 4; the pre-surgery visit (1–2 days prior to cytoreductive surgery) and the post-surgery visit (1 ± 1 day)

| Group | Cytokine | p-value (unadjusted) | p-value (adj. BH) | Mean change | SD |
| --- | --- | --- | --- | --- | --- |
| Inflammatory cytokines | IL-1β | 0.004 | *0.008* | -0.64 | 0.58 |
|  | IL-2 | 0.000 | *0.001* | -1.24 | 0.73 |
|  | IL-6 | 0.000 | *0.000* | 3.24 | 1.08 |
|  | IL-8 | 0.121 | 0.186 | 0.27 | 0.53 |
|  | IL-12 | 0.253 | 0.364 | -0.61 | 1.65 |
|  | IL-15 | 0.703 | 0.703 | 0.20 | 1.72 |
|  | IL-17 | 0.013 | *0.023* | -0.88 | 0.97 |
|  | TNF-α | 0.299 | 0.382 | -0.11 | 0.33 |
| Anti-inflammatory cytokines | IL1Ra | 0.004 | *0.008* | 1.71 | 1.51 |
|  | IL-4 | 0.002 | *0.004* | -0.59 | 0.46 |
|  | IL-5 | 0.540 | 0.591 | -0.19 | 1.00 |
|  | IL-9 | 0.343 | 0.415 | -0.21 | 0.70 |
|  | IL-13 | 0.000 | *0.001* | -0.78 | 0.45 |
| Growth factors | FGF-β | 0.001 | *0.003* | -0.44 | 0.31 |
|  | G-CSF | 0.438 | 0.504 | -0.15 | 0.62 |
|  | GM-CSF | 0.120 | 0.186 | -0.35 | 0.68 |
|  | IL-7 | 0.000 | *0.001* | -0.66 | 0.39 |
|  | PDGF-BB | 0.685 | 0.703 | 0.05 | 0.36 |
| Chemokines | Eotaxin | 0.000 | *0.000* | -0.70 | 0.35 |
|  | IP-10 | 0.000 | *0.000* | -0.82 | 0.29 |
|  | MCP-1 | 0.002 | *0.004* | 0.58 | 0.46 |
|  | MIP-1α | 0.002 | *0.004* | -0.50 | 0.38 |
|  | MIP-1β | 0.285 | 0.382 | -0.07 | 0.21 |

adj. BH: P-value adjusted with the Benjamini-Hochberg method. Negative mean change means that the pre-test value has a lower mean than the post-test value. P-values ≤0.05 is considered significant. Significant values are indicated in italic font.

**Supplementary Table S6:** Changes in cytokine levels between visit 1 and 3; from inclusion and prior to primary cytoreductive surgery (before and after the laparoscopic procedure) (n = 11)

| Group | Cytokine | p-value (unadjusted) | p-value (adj. BH) | Mean change | SD |
| --- | --- | --- | --- | --- | --- |
| Inflammatory cytokines | IL-1β | 0.456 | 0.656 | 0.06 | 0.25 |
|  | IL-2 | 0.597 | 0.808 | 0.14 | 0.88 |
|  | IL-6 | 0.255 | 0.534 | -0.21 | 0.61 |
|  | IL-8 | 0.817 | 0.853 | 0.02 | 0.31 |
|  | IL-12 | 0.181 | 0.463 | 0.63 | 1.53 |
|  | IL-15 | 0.440 | 0.881 | -0.07 | 1.51 |
|  | IL-17 | 0.260 | 0.534 | 0.09 | 0.26 |
|  | TNF-α | 0.019 | 0.112 | 0.14 | 0.17 |
| Anti-inflammatory cytokines | IL1Ra | 0.279 | 0.534 | 0.28 | 0.84 |
|  | IL-4 | 0.790 | 0.853 | -0.01 | 0.16 |
|  | IL-5 | 0.026 | 0.119 | 0.74 | 1.00 |
|  | IL-9 | 0.426 | 0.853 | 0.03 | 0.55 |
|  | IL-13 | 0.367 | 0.602 | 0.11 | 0.39 |
| Growth factors | FGF-β | 0.117 | 0.384 | 0.17 | 0.35 |
|  | G-CSF | 0.010 | 0.073 | 0.27 | 0.29 |
|  | GM-CSF | 0.737 | 0.853 | -0.05 | 0.50 |
|  | IL-7 | 0.779 | 0.853 | 0.02 | 0.26 |
|  | PDGF-BB | 0.365 | 0.602 | -0.07 | 0.26 |
| Chemokines | Eotaxin | 0.456 | 0.656 | -0.03 | 0.15 |
|  | IP-10 | 0.152 | 0.437 | 0.05 | 0.12 |
|  | MCP-1 | 0.101 | 0.384 | 0.09 | 0.17 |
|  | MIP-1α | 0.004 | *0.047* | 0.28 | 0.27 |
|  | MIP-1β | 0.004 | *0.047* | 0.11 | 0.10 |

Comparisons were made between time of inclusion (within 14 days of treatment) and prior to primary surgery (14 ± 7 days). adj. BH = P value adjusted with the Benjamini-Hochberg method. P-values ≤0.05 is considered significant. Significant values are indicated in italic font.

**Supplementary Table S7**: Changes in cytokine levels between visit 3 and visit 6: pre-surgery and pre-chemo.

| Group | Cytokine | p-value (unadjusted) | p-value (adj. BH) | Mean change | SD |
| --- | --- | --- | --- | --- | --- |
| Inflammatory cytokines | IL-1β | 0.481 | 0.922 | -0.09 | 0.41 |
|  | IL-2 | 0.024 | 0.553 | -0.64 | 0.80 |
|  | IL-6 | 0.772 | 0.923 | -0.10 | 1.07 |
|  | IL-8 | 0.213 | 0.711 | -0.21 | 0.52 |
|  | IL-12 | 0.216 | 0.711 | 0.78 | 1.96 |
|  | IL-15 | 0.903 | 0.923 | -0.09 | 2.49 |
|  | IL-17 | 0.815 | 0.923 | -0.04 | 0.50 |
|  | TNF- α | 0.593 | 0.923 | -0.06 | 0.35 |
| Anti-inflammatory cytokines | IL1Ra | 0.605 | 0.923 | 0.24 | 1.51 |
|  | IL-4 | 0.162 | 0.711 | -0.16 | 0.35 |
|  | IL-5 | 0.844 | 0.923 | -0.06 | 1.04 |
|  | IL-9 | 0.595 | 0.923 | 0.08 | 0.48 |
|  | IL-13 | 0.923 | 0.923 | -0.01 | 0.48 |
| Growth factors | FGF- β | 0.728 | 0.923 | -0.08 | 0.71 |
|  | G-CSF | 0.328 | 0.838 | -0.10 | 0.31 |
|  | GM-CSF | 0.147 | 0.711 | 0.49 | 1.04 |
|  | IL-7 | 0.459 | 0.922 | -0.07 | 0.31 |
|  | PDGF-BB | 0.459 | 0.922 | -0.11 | 0.46 |
| Chemokines | Eotaxin | 0.278 | 0.800 | 0.08 | 0.23 |
|  | IP-10 | 0.202 | 0.711 | -0.21 | 0.51 |
|  | MCP-1 | 0.134 | 0.711 | -0.14 | 0.28 |
|  | MIP-1α | 0.881 | 0.923 | -0.02 | 0.34 |
|  | MIP-1β | 0.790 | 0.923 | 0.02 | 0.22 |

Comparisons were made between cytokine levels in pre-surgery samples, collected 1–2 days prior to cytoreductive surgery, and samples collected after surgery, prior to initiation of chemotherapy (after 42 ± 14 days). Adj. BH = P value adjusted with the Benjamini-Hochberg method. P-values ≤0.05 is considered significant. Significant values are indicated in italic font.

**Supplementary Table S8**: Changes in cytokine levels between visit 4 and 5; post-surgery and day 12 (± 3 days) after the primary cytoreductive surgery (n = 8)

| Group | Cytokine | p-value (unadjusted) | p-value (adj. BH) | Mean change | SD |
| --- | --- | --- | --- | --- | --- |
| Inflammatory cytokines | IL-1β | 0.136 | 0.220 | 0.55 | 0.93 |
|  | IL-2 | 0.002 | *0.008* | 1.06 | 0.61 |
|  | IL-6 | 0.000 | *0.001* | -2.72 | 0.79 |
|  | IL-8 | 0.392 | 0.475 | -0.23 | 0.71 |
|  | IL-12 | 0.150 | 0.220 | 1.03 | 1.80 |
|  | IL-15 | 0.438 | 0.504 | 0.48 | 1.65 |
|  | IL-17 | 0.107 | 0.190 | 0.70 | 1.08 |
|  | TNF-α | 0.650 | 0.680 | 0.08 | 0.47 |
| Anti-inflammatory cytokines | IL1Ra | 0.178 | 0.228 | -0.64 | 1.21 |
|  | IL-4 | 0.007 | *0.020* | 0.54 | 0.41 |
|  | IL-5 | 0.740 | 0.740 | -0.14 | 1.14 |
|  | IL-9 | 0.153 | 0.220 | 0.34 | 0.59 |
|  | IL-13 | 0.015 | *0.038* | 0.87 | 0.77 |
| Growth factors | FGF-β | 0.086 | 0.165 | 0.25 | 0.36 |
|  | G-CSF | 0.002 | *0.008* | 0.43 | 0.24 |
|  | GM-CSF | 0.166 | 0.224 | 0.52 | 0.95 |
|  | IL-7 | 0.003 | *0.010* | 0.64 | 0.40 |
|  | PDGF-BB | 0.517 | 0.567 | 0.12 | 0.50 |
| Chemokines | Eotaxin | 0.001 | *0.004* | 0.73 | 0.35 |
|  | IP-10 | 0.007 | *0.020* | 0.66 | 0.50 |
|  | MCP-1 | 0.026 | 0.060 | -0.51 | 0.52 |
|  | MIP-1α | 0.000 | *0.003* | 0.75 | 0.31 |
|  | MIP-1β | 0.058 | 0.122 | 0.18 | 0.22 |

These values are not included in the multivariate analysis as the number of samples was too small. Adj. BH = P value adjusted with the Benjamini-Hochberg method. P-values ≤0.05 is considered significant. Significant values are indicated in italic font.

**Supplementary Table S9:** Changes in cytokine levels between visit 1 and 2; associated with the diagnostic laparoscopic procedure (n = 20) (from inclusion to post-laparoscopy visit)

| Group | Cytokine | p-value (unadjusted) | p-value (adj. BH) | Mean change | SD |
| --- | --- | --- | --- | --- | --- |
| Inflammatory cytokines | IL-1β | 0.017 | 0.064 | -0.26 | 0.45 |
|  | IL-2 | 0.062 | 0.158 | -0.56 | 1.27 |
|  | IL-6 | 0.031 | 0.101 | 0.59 | 1.13 |
|  | IL-8 | 0.986 | 0.986 | 0.00 | 0.49 |
|  | IL-12 | 0.730 | 0.840 | -0.12 | 1.58 |
|  | IL-15 | 0.043 | 0.124 | -0.84 | 1.73 |
|  | IL-17 | 0.115 | 0.203 | -0.11 | 0.31 |
|  | TNF-α | 0.113 | 0.217 | -0.07 | 0.20 |
| Anti-inflammatory cytokines | IL1Ra | 0.793 | 0.869 | -0.07 | 1.15 |
|  | IL-4 | 0.000 | *<0.001* | -0.35 | 0.28 |
|  | IL-5 | 0.972 | 0.986 | 0.01 | 1.37 |
|  | IL-9 | 0.695 | 0.842 | -0.04 | 0.44 |
|  | IL-13 | 0.100 | 0.208 | -0.23 | 0.58 |
| Growth factors | FGF-β | 0.232 | 0.381 | -0.14 | 0.50 |
|  | G-CSF | 0.362 | 0.521 | -0.07 | 0.33 |
|  | GM-CSF | 0.093 | 0.215 | -0.27 | 0.69 |
|  | IL-7 | 0.001 | *0.008* | -0.22 | 0.26 |
|  | PDGF-BB | 0.595 | 0.761 | 0.03 | 0.27 |
| Chemokines | Eotaxin | 0.000 | *<0.001* | -0.27 | 0.20 |
|  | IP-10 | 0.000 | *0.001* | -0.23 | 0.21 |
|  | MCP-1 | 0.011 | *0.049* | -0.34 | 0.53 |
|  | MIP-1α | 0.376 | 0.508 | -0.07 | 0.35 |
|  | MIP-1β | 0.362 | 0.555 | -0.03 | 0.17 |

Cytokine changes between the time of inclusion (within 14 days of the laparoscopic procedure) and the first post-laparoscopy visit (1 ± 1 days) were analyzed. Adj. BH = P value adjusted with the Benjamini-Hochberg method. P-values ≤0.05 is considered significant. Significant values are indicated in italic font.

**Supplementary Table S10:** Changes in cytokine levels between visit 1 and 7; inclusion and the end of study (n = 17)

| Group | Cytokine | p-value (unadjusted) | p-value (adj. BH) | Mean change | SD |
| --- | --- | --- | --- | --- | --- |
| Inflammatory cytokines | IL-1β | 0.972 | 0.972 | 0.00 | 0.44 |
|  | IL-2 | 0.003 | *0.011* | -0.97 | 1.14 |
|  | IL-6 | 0.001 | *0.006* | -1.07 | 1.11 |
|  | IL-8 | 0.000 | *0.002* | -0.62 | 0.53 |
|  | IL-12 | 0.875 | 0.978 | 0.05 | 1.39 |
|  | IL-15 | 0.023 | 0.058 | -0.86 | 1.41 |
|  | IL-17 | 0.674 | 0.894 | -0.05 | 0.44 |
|  | TNF-α | 0.002 | *0.009* | -0.24 | 0.27 |
| Anti-inflammatory cytokines | IL1Ra | 0.699 | 0.894 | -0.14 | 1.43 |
|  | IL-4 | 0.935 | 0.978 | -0.01 | 0.32 |
|  | IL-5 | 0.902 | 0.978 | 0.05 | 1.52 |
|  | IL-9 | 0.677 | 0.894 | -0.04 | 0.34 |
|  | IL-13 | 0.127 | 0.266 | 0.16 | 0.42 |
| Growth factors | FGF-β | 0.582 | 0.893 | -0.06 | 0.44 |
|  | G-CSF | 0.334 | 0.590 | -0.09 | 0.36 |
|  | GM-CSF | 0.850 | 0.978 | -0.03 | 0.63 |
|  | IL-7 | 0.232 | 0.445 | -0.09 | 0.31 |
|  | PDGF-BB | 0.000 | *0.000* | -1.04 | 0.45 |
| Chemokines | Eotaxin | 0.539 | 0.886 | 0.04 | 0.24 |
|  | IP-10 | 0.000 | *0.003* | -0.50 | 0.46 |
|  | MCP-1 | 0.007 | *0.020* | -0.38 | 0.51 |
|  | MIP-1α | 0.067 | 0.154 | -0.19 | 0.40 |
|  | MIP-1β | 0.004 | *0.012* | -0.17 | 0.20 |

Adj. BH = P-value adjusted with the Benjamini-Hochberg method. P-values ≤0.05 is considered significant. Significant values are indicated in italic font.


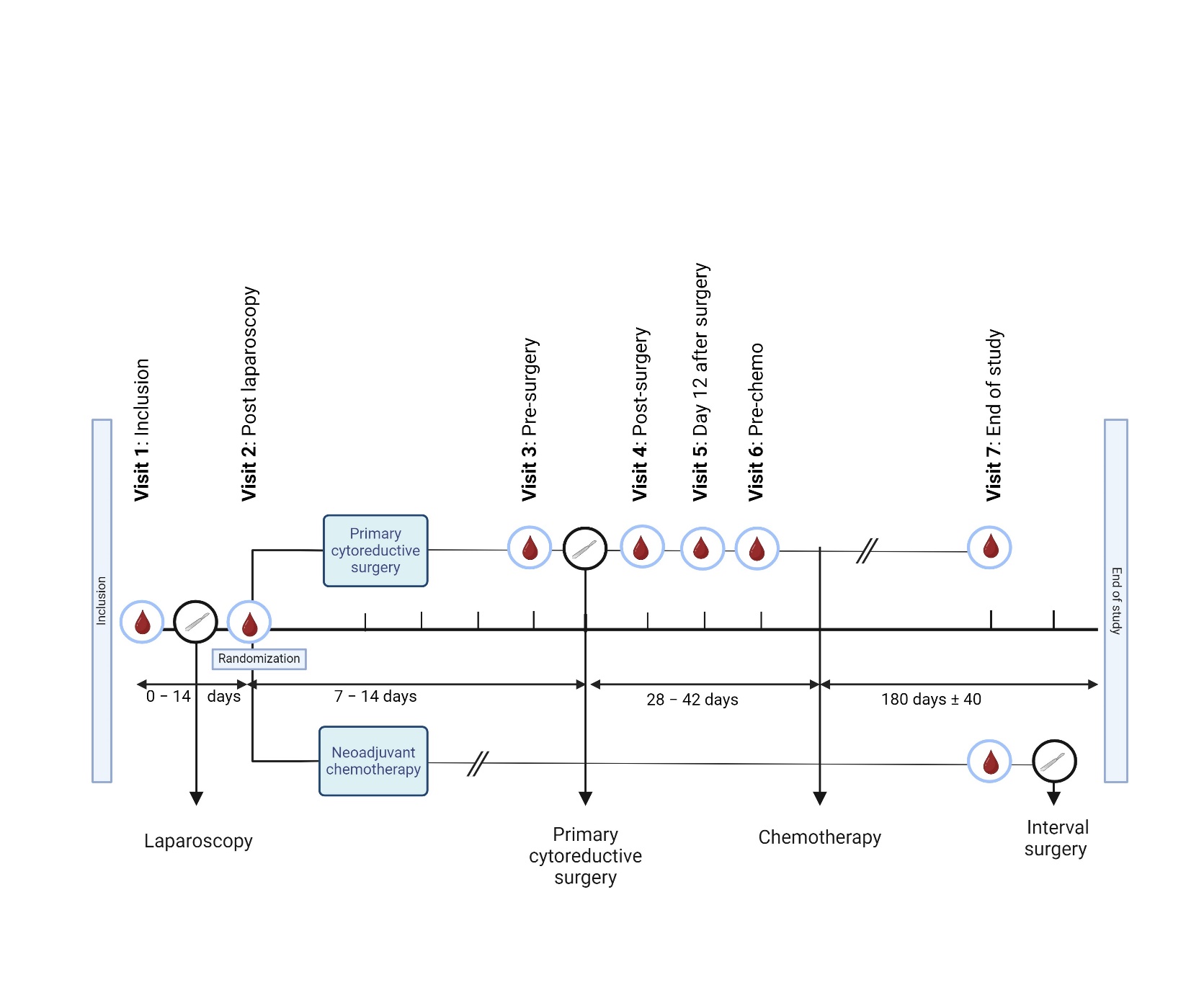


**Supplementary Figure S1:** Study protocol and overview of the different timepoints. **Supplementary Table S4** shows changes in cytokine levels between the pre-surgery visit (1-2 days prior to cytoreductive surgery) and the post-surgery visit (1 ± 1 days after cytoreductive surgery). Marked changes were found for 13 cytokines in all four functional groups: increased levels of the inflammatory cytokine IL-6 and IL-8, the anti-inflammatory cytokine IL-1Ra, and the chemokine MCP-1 were identified, while levels were reduced for the chemokines IP-10, eotaxin, and MIP-1α, inflammatory cytokines IL-2, IL-1β, and IL-17, anti-inflammatory cytokines IL-13 and IL-4, and the growth factors IL-7 and FGF-β.**Supplementary Table S5** shows changes in cytokine levels from inclusion and prior to primary cytoreductive surgery (before and after the laparoscopic procedure). Marked changes were only found with increased levels of two chemokines: MIP-1 α and MIP-1β. **Supplementary Table S6** shows changes in cytokine levels before and after primary cytoreductive surgery. Comparison of cytokine levels 1-2 days prior to cytoreductive surgery and samples collected pripr to initiation of chemotherapy (42 ± 14 days after cytoreductive surgery). No significant changes in cytokine expression were discovered between these two visits. **Supplementary Table S7** shows changes in cytokine levels immediately after surgery (0+2 days) and day 12 ((± 3 days) after the primary cytoreductive surgery. Marked changes was found with increased levels of the inflammatory cytokine IL-2, reduced levels of the inflammatory cytokine IL-6, increased levels of IL-4 and IL-13, increased levels of the growth factor G-CSF and IL-7 and increased levels of the chemokines eotaxin, IP-10 and MIP-1 β. **Supplementary Table S8** shows changes in cytokine levels associated with the diagnostic laparoscopic procedure. Marked changes were found with increased levels of the anti-inflammatory cytokine IL-4, The growth factor IL-7, and the chemokines eotaxin, IP-10 and MCP-1. **Supplementary Table S9** shows changes in cytokines levels associated with cytoreductive surgery in the Immune High subgroup and Immune Low subgroup. The Immune High subgroup exhibited a more extensive and seemingly more robust immune response following the surgical treatment, characterized by diminished levels of growth factors, anti-inflammatory cytokines, and various other inflammatory cytokines. The Immune Low subgroup only showed marked changes were elevated levels of the chemokines eotaxin and IP-10. In **Supplementary Table S3** it is also shown that the Immune High group showed a significant change in cytokine levels in response to the diagnostic laparoscopy with a reduction in the levels of the anti-inflammatory IL-4 and the chemokines IP-10 and eotaxin. There was an evident difference in the overall treatment response between the Immune High and Immune Low patients, with a broad and significant response observed only in the Immune High group, which showed reduced levels of the inflammatory cytokines IL-6, IL-8, IL-15 and TNF-α the growth factors PDGF-BB and FGF-β, and the chemokines IP-10 and MIP-1β. In contrast, no significant cytokine changes occurred from inclusion to the end of treatment in the Immune Low group. **Supplementary Table S10** shows changes in cytokine levels from inclusion to the end of study visit. There was an overall reduction in cytokines mainly mediated by the growth factor PDGF-BB, inflammatory cytokines IL-6, IL-8, IL-2 and TNF-α, and the chemokines IP-10 and MIP1β.


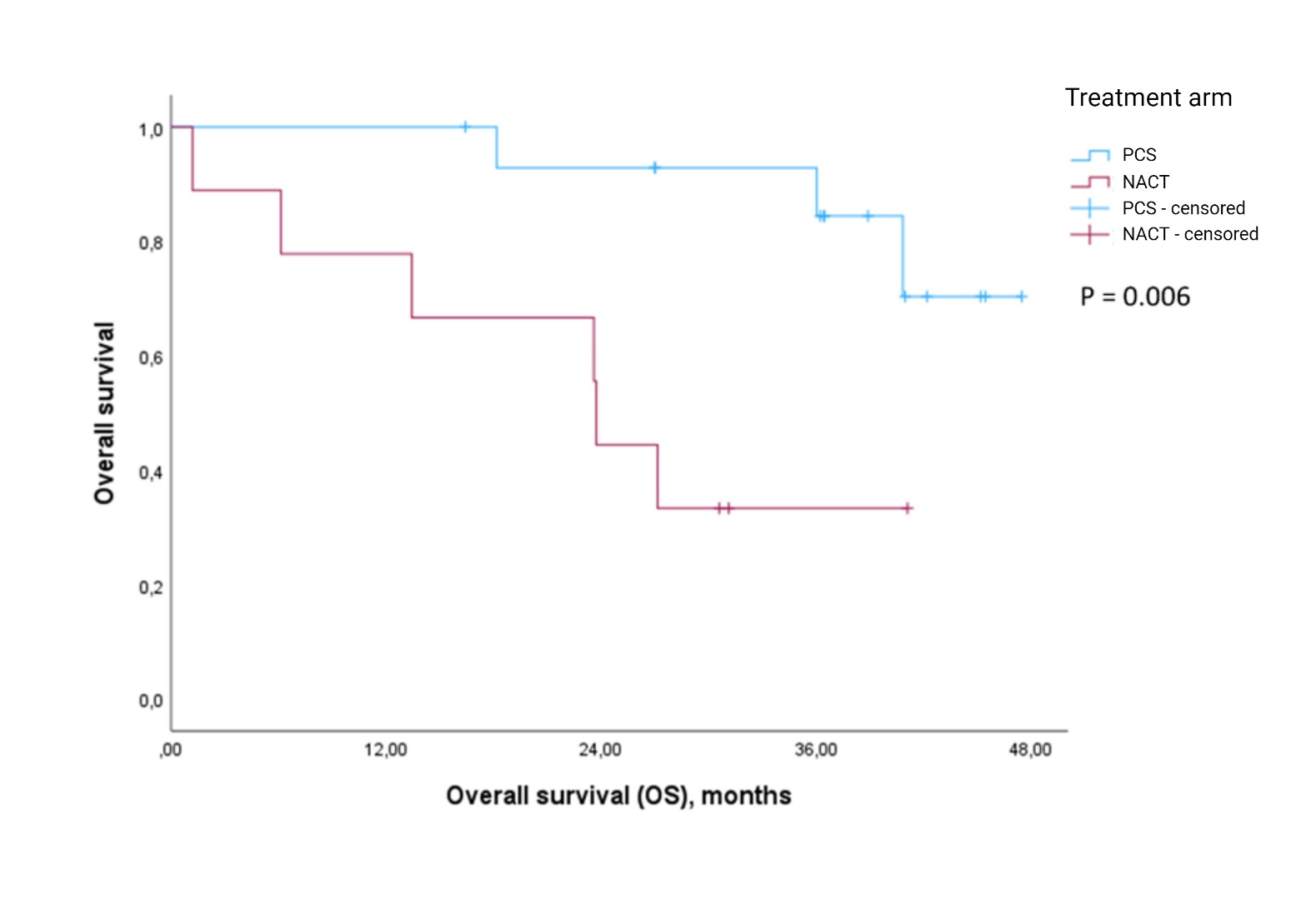


**Supplementary Figure S2:** Kaplan Meier survival curve (PCS; Primary cytoreductive surgery vs NACT; neoadjuvant chemotherapy) (n = 24).


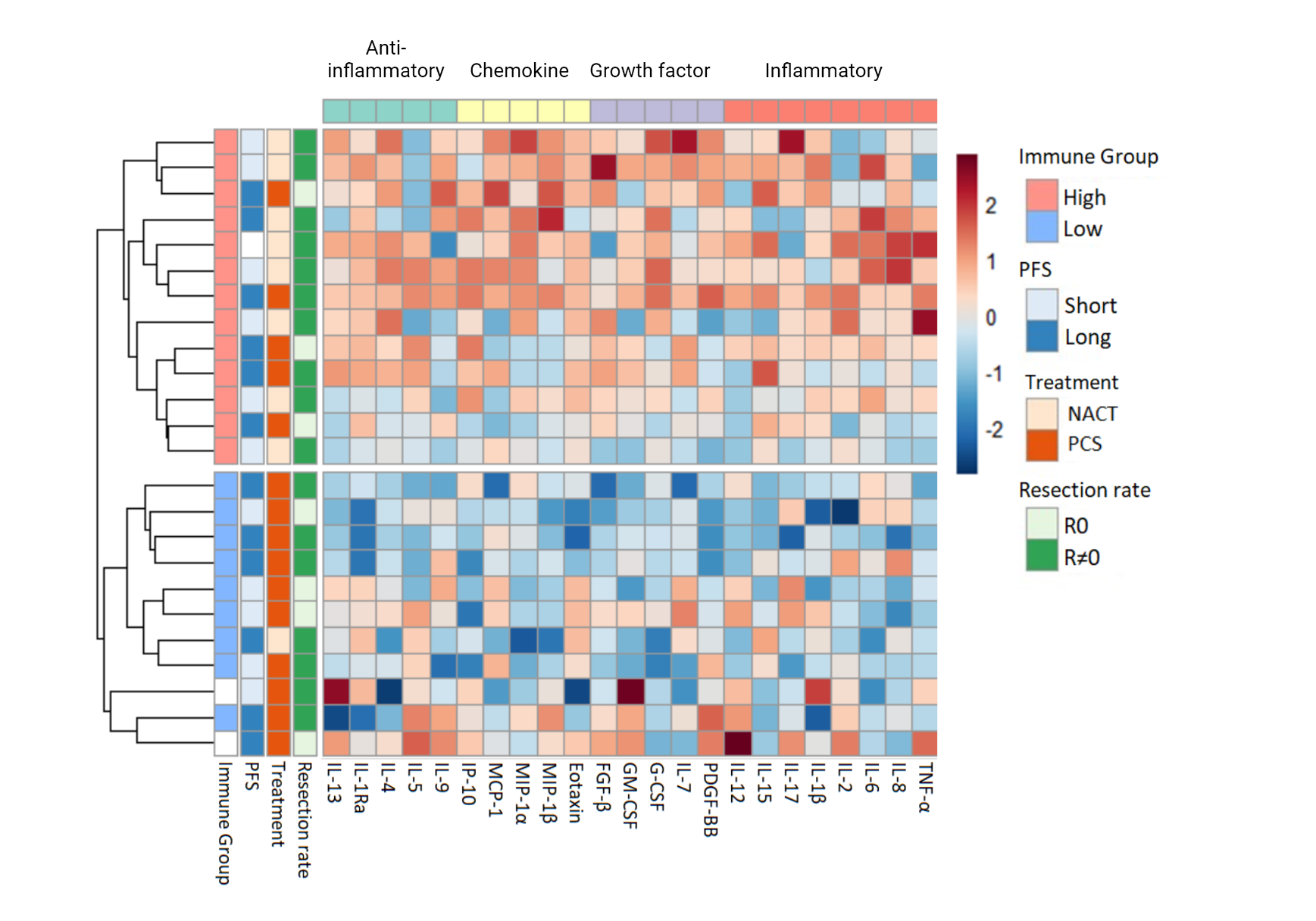


**Supplementary Figure S3:** Heatmap with unsupervised hierarchical clustering of patients (n = 24) at inclusion, before treatment initiation

Each row represents one patient. The blocks are color coded according to the predefined relevant subgroups (right). The cytokine classes are shown at the top. The color scale represents the serum concentrations of cytokines, with lower concentrations shown in blue and higher concentrations shown in red. The color scale is relative and scaled for inter-individual differences between patients for each cytokine.
